# Perception and lived experience of movement in patients with fibromyalgia: a qualitative systematic review with meta-synthesis and meta-summary

**DOI:** 10.1007/s10067-026-08005-1

**Published:** 2026-02-25

**Authors:** Matteo Cioeta, Martina Sitzia, Michele Marelli, Silvia Bargeri, Giuseppe Giovannico, Leonardo Pellicciari, Germano Guerra, Mauro Crestani, Alvisa Palese, Chad Cook, Giacomo Rossettini

**Affiliations:** 1 Department of Medicine and Health Science “Vincenzo Tiberio”, University of Molise c/o Cardarelli Hospital, Campobasso, Italy; 2 Department of Medico-Surgical Sciences and Biotechnologies, “Sapienza” University of Rome-Polo Pontino, Corso Della Repubblica 79, Latina, 04100 Italy; 3 Unit of Clinical Epidemiology, IRCCS Ospedale Galeazzi Sant’Ambrogio, Milan, Italy; 4 IRCCS Istituto Delle Scienze Neurologiche Di Bologna, Bologna, Italy; 5 Department of Neurosciences, Biomedicine and Movement Sciences, University of Verona, Verona, Italy; 6 Department of Medical Sciences, University of Udine, Udine, Italy; 7 Department of Orthopaedics, Duke University, Durham, NC USA; 8 Duke Clinical Research Institute, Duke University, Durham, NC USA; 9 Department of Population Health Sciences, Duke University, Durham, NC USA; 10 School of Physiotherapy, University of Verona, Verona, Italy; 11 Department of Physiotherapy, Faculty of Sport Sciences, Universidad Europea de Madrid, Villaviciosa de Odón, Spain

**Keywords:** Barriers, Exercise, Facilitators, Fibromyalgia, Movement, Qualitative synthesis

## Abstract

**Objective:**

To systematically synthesize and summarize qualitative findings on how adults with fibromyalgia perceive, negotiate, and sustain exercise and everyday movement.

**Design:**

Systematic review of qualitative studies with meta-synthesis and meta-summary.

**Literature search:**

We searched CINAHL, EMBASE, PsycINFO, MEDLINE, Scopus, SPORTDiscus, and Web of Science until October 2025, supplemented by berry-picking techniques and gray literature.

**Study selection criteria:**

We included qualitative primary studies or mixed-methods studies that clearly reported qualitative data; involved adults (≥ 18 years) with fibromyalgia; and explored experiences of movement, physical activity, or exercise.

**Data synthesis:**

Sandelowski and Barroso’s methodology was used for study classification, meta-synthesis, and meta-summary. Methodological quality was appraised with Critical Appraisal Skills Programme and Mixed Methods Appraisal Tool. The Confidence in the Evidence from Reviews of Qualitative Research approach evaluated certainty.

**Results:**

Thirteen studies (*N* = 213, 204 women; 19–82 years) were included. Four hundred thirty-two statements were extracted, synthesized into 15 categories, and grouped into four themes: (1) past experiences of movement; (2) movement during daily life: strategies, adaptations, and effects; (3) barriers to movement: personal, environmental, and relational; (4) facilitators to movement: peer support, empathic relationships, and f guide. Inter-study frequency effect sizes were highest for “Positive effects” (77%) and “Altered body perception” (69%) and lowest for “Standardized, not personalized plan” (31%). Overall confidence in the findings was moderate.

**Conclusion:**

For individuals with fibromyalgia, movement is experienced as both beneficial and risky. Patient-centered rehabilitation could validate pacing, formalize existing self-management strategies, and offer tailored, supported pathways to sustainable activity.

**Supplementary Information:**

The online version contains supplementary material available at 10.1007/s10067-026-08005-1.

## Introduction

Fibromyalgia (FM) is a complex syndrome characterized by chronic widespread musculoskeletal pain, non-restorative sleep, fatigue, mood disturbance, and cognitive difficulties [15]. Pain is persistent, often described as stabbing or burning, and fluctuates in intensity and distribution; central sensitization phenomena such as hyperalgesia and allodynia are common [12]. Comorbid anxiety and depression can amplify catastrophizing and rumination and correlate with cognitive impairment [34]. Global prevalence ranges from 1.3% to 8%, peaking among women aged 20–55 years, in whom FM is the most common musculoskeletal pain diagnosis [1].

Current guidelines emphasize a personalized, multimodal approach combining education, cognitive-behavioral therapy, exercise, and where necessary, pharmacotherapy [36, 54]. Exercise is a key non-pharmacological treatment for FM [47]; a multicomponent plan that integrates aerobic, resistance, flexibility, and mind–body elements reduces pain and improves physical function and quality of life [5]. Training should be individualized and progressed gradually in line with preferences, comorbidities, and fitness levels [9, 44]. Nevertheless, adherence is often undermined by fear of symptom flares and overwhelming fatigue [56].

Individually reviewed qualitative research highlights heterogeneous and, at times, ambivalent perceptions of movement and exercise in FM [29, 30, 41, 53]. Personal beliefs shape engagement as some individuals experience movement as disabling, whereas others view it as a source of psychosocial well-being [53]. Pain and fatigue commonly act as barriers, while group activities, professional guidance, and graded pacing facilitate participation [30]. Walking, in particular, elicits mixed representations ranging from discomfort to an opportunity for progress and social interaction [53]. Regular exercisers often emphasize the importance of physiotherapist support in tailoring intensity and addressing concerns [27, 39].

Existing qualitative syntheses in FM emphasize lived experience and diagnostic journeys, not exercise or everyday movement as primary phenomena [7, 39, 56], while reviews of primary-care encounters and stigma contextualize motivation but do not analyze activity behaviors [16, 18, 26, 31]. These gaps justify a dedicated qualitative synthesis on how adults with FM perceive and negotiate exercise and daily movement to inform patient-centered rehabilitation. The aim of this study is to synthesize the qualitative evidence on how adults with FM perceive exercise and everyday movement strategies. We propose that a synthesis could identify patient-reported barriers and facilitators influencing adherence and inform patient-centered rehabilitation plans.

## Methods

### Study design

We conducted a systematic review of qualitative studies with meta-synthesis and meta-summary using Sandelowski and Barroso’s methodology [50] that encompasses the following: (1) formulating the research question, (2) systematically searching and extracting data for analysis, (3) appraising the quality of included studies, (4) classifying studies, and (5) synthesizing data through meta-synthesis and meta-summary. The approach facilitates collecting different patients’ experiences and perspectives across various contexts and reinterpreting findings into new theoretical models (24). Our review was prospectively registered in the Open Science Framework Registries database in September 2025 (https://osf.io/vryg6) and prepared following international reporting standards [32, 40, 58].

### Search strategy

A systematic search was conducted across seven electronic databases (CINAHL, EMBASE, PsycINFO, MEDLINE, Scopus, SPORTDiscus, and Web of Science) from their inception to October 2025. All search terms were identified and organized using the SPIDER (Sample, Phenomenon of Interest, Design, Evaluation, Research type) framework for qualitative research [20] (Supplementary File 1).

We used a “berry picking” approach to ensure a comprehensive search [50]. This included techniques such as footnote chasing, citation searching, hand searching, journal run, author searching, and gray literature (e.g., master’s theses and PhD dissertations). The search strategy was developed in consultation with a health information specialist from a medical library to enhance accuracy and reliability. Detailed combinations of keywords and Thesaurus terms used for all database searches are provided in Supplementary File 2.

### Eligibility criteria

Inclusion criteria were as follows: (1) qualitative primary studies or mixed-methods studies that clearly identified participants and findings derived from qualitative methods; (2) studies published in any language; (3) involving participants aged ≥ 18 years with FM; and (4) exploring participants’ experiences of movement, physical activity, or exercise in daily life. Exclusion criteria were as follows: (1) qualitative or mixed-methods studies without clear identification of participants and findings derived from qualitative methods and (2) studies involving participants with other chronic pain conditions.

For this review, movement/physical activity was defined as any bodily activity involving skeletal muscles and energy expenditure, encompassing (i) structured exercise and planned physical activity (e.g., aerobic, strengthening, flexibility, or multimodal programs), (ii) incidental/lifestyle physical activity (e.g., walking for transport and stair climbing), and (iii) everyday functional movement and activities of daily living (e.g., household tasks, self-care, and occupational activities). We also considered movement experiences described within rehabilitation or exercise-therapy contexts when the qualitative focus was on participants’ lived experiences of moving (e.g., beliefs, emotions, meaning-making, strategies, and barriers/facilitators), rather than solely on intervention efficacy.

Given common comorbidities, studies were not excluded based on comorbid conditions when participants with FM were clearly identifiable, and their movement-related qualitative data could be extracted separately. Mixed-population studies (e.g., “chronic pain” samples) were included only when FM-specific data were clearly and adequately reported, defined as at least one of the following: (a) verbatim quotations explicitly attributed to participants with FM; (b) analytic findings/themes reported separately for the FM subgroup; or (c) stratified presentation of results by diagnosis allowing unambiguous extraction. Studies were excluded when quotations were not linked to diagnosis and findings were presented only at an aggregated mixed-sample level, preventing confident attribution to FM.

### Study selection

Records retrieved from the different databases were imported into Rayyan Qatar Computing Research Institute online software (https://www.rayyan.ai/) [45]. After duplicates were removed, two reviewers (MC and GeG) independently performed title and abstract screening, followed by full-text screening, based on the inclusion/exclusion criteria. Disagreements were resolved through consultation with the overall research group.

### Data extraction

Data extraction from the included studies was conducted independently by two authors (MM and MS). A summary table was used to synthesize the extracted data, detailing the first author’s name and year of publication, country, aim, participants, data collection, analysis, and qualitative findings summarizing the participants’ experiences. When data specifically related to patients with FM were clearly and adequately reported in the manuscripts and/or supplemental materials of the included studies, these data were extracted and incorporated into the review. Findings were then categorized based on the extent to which the researcher transformed the raw data for analysis and synthesis. The classification system, following Sandelowski and Barroso’s guideline [50], included the following: (1) thematic surveys (e.g., the latent pattern of themes discerned from data), (2) conceptual/thematic descriptions (e.g., concepts or themes developed in situ), and (3) interpretive explanations (e.g., fully integrated explanations of the phenomenon). Any disagreements were resolved through consensus with the research group.

### Critical appraisal

The Critical Appraisal Skills Programme (CASP) tool [10] was used to assess the methodological quality of the included qualitative studies. Low quality was not used as a criterion for exclusion. Instead, the evaluation of such studies was incorporated to strengthen the rigor of this systematic review [49–51]. The CASP tool includes 10 questions []. For items one through nine, the CASP adopts a 3-point scoring system: “Yes” for well-described aspects, “No” for aspects not described, and “Can’t tell” for unclear or insufficiently detailed elements. Item 10 is an open-ended question assessing the importance of the study (e.g., whether it is considered valuable or not). To allow consistent item-level counting and comparison across studies, Item 10 (“How valuable is the research?”), which is typically assessed narratively, was operationalized as a binary judgement. We coded “Yes” when the study was considered valuable to the review aims (e.g., offering clinically meaningful insights and/or conceptually rich data relevant to everyday movement and exercise in FM) and “No” when it was considered not valuable; “Can’t tell” was used when the report lacked sufficient information to make this judgement. The CASP tool has been applied in qualitative synthesis on musculoskeletal pain conditions [21, 22].

The Mixed Methods Appraisal Tool (MMAT) was used to assess the methodological quality of any mixed-methods studies we included in the review [25]. The tool consists of two initial screening questions and 25 criteria, five of which are specific to each study design category. Each criterion is rated using a 3-point scale: “Yes” (criterion met), “No” (criterion not met), and “Can’t tell” (insufficient information to make a judgment). Two authors (MC and GiG) independently evaluated the quality of the included articles using a similar system for resolving disagreements, as previously described.

### Confidence assessment

The Confidence in the Evidence from Reviews of Qualitative Research (GRADE-CERQual) [33] approach was adopted to evaluate the certainty of the review findings and provides a systematic and transparent approach of assessment based on four components: (1) methodological limitations [42], (2) relevance [43], (3) adequacy [24], and (4) coherence [19]. Two reviewers (MC and GR) independently assessed the confidence in individual findings from the qualitative evidence synthesis with the aforementioned method of handling disagreements. Methodological limitations were informed by the CASP/MMAT appraisals. Relevance was assessed based on the fit between the contributing populations and contexts and the review question. Adequacy was judged by the volume and richness of the supporting data contributing to each finding (e.g., number of contributing studies and the depth of descriptive/interpretive data available). Coherence was judged by the extent to which the data supporting each finding formed a clear and well-supported pattern within and across studies, including whether any apparent variation could be plausibly explained. Theme-specific justifications, including brief illustrative examples, are summarized in Table 5, with extended material provided in Supplementary File 3.

### Data synthesis and analysis

Meta-synthesis and meta-summary were conducted following the methodological steps outlined by Sandelowski and Barroso [50] and were performed independently by two authors (MM and MS) (Supplementary File 4). In this review, the unit of extraction, referred to as a “statement,” consisted of a verbatim participant quotation reported in the included primary studies. Quotations were extracted primarily from the Results/Findings sections, including quotes presented in the main text as well as in tables/boxes/figures and supplementary materials; text outside the Results/Findings was considered only when it contained clearly identifiable verbatim participant data. Two reviewers (MM and MS) independently extracted and coded the quotations. Coding was conducted inductively at the semantic level using line-by-line/open coding, and a shared codebook was iteratively refined through constant comparison across studies. Codes were then clustered into higher-order categories, which were merged/split as needed through iterative discussion until stable categories were agreed upon; these categories were subsequently abstracted into the final overarching themes. Coding disagreements (eligibility of a quotation for extraction and/or its coding/category allocation) were resolved through item-by-item discussion, with adjudication by a third reviewer (MC) when needed.

The estimation of two metrics was performed: (1) the inter-study frequency effect size, which refers to the prevalence of themes, calculated as (number of studies containing a theme/total number of studies) × 100, and (2) the intra-study intensity effect size, which refers to the concentration of themes within each report, calculated as (number of themes in the study/total number of themes) × 100. Disagreements were resolved through consultation/consensus with the research group [50]. Detailed themes and statements of the meta-synthesis process were provided in Supplementary File 5.

### Credibility strategies

To ensure the validity, rigor, and trustworthiness of the meta-synthesis and meta-summary, a multidisciplinary team of experts actively reviewed and critiqued the study procedures and results throughout the process [50]. Our multidisciplinary team comprised physiotherapists, nurse researchers, academics, and methodologists with expertise in managing FM and qualitative research synthesis. Although the diverse clinical and methodological experiences may have influenced the collection and interpretation of data, the blend of skills and professional perspectives, combined with a collaborative and reflective approach, enriched the interpretation of the findings, thereby providing an authentic representation of patients’ experiences. These strategies [25], combined with the background diversity of team members, increased the transparency of the process and findings. Any discrepancies were resolved through consultation and consensus within the research group. Details of these strategies are available in Supplementary File 6.

In addition, a stakeholder with personal experience of FM was involved [8, 25] in reviewing, participating in, and discussing the protocol, thereby validating its relevance and impact and confirming the importance of the review. The patient agreed with the final elements of structure, affirming the methodological rigor. Following Cochrane’s guidelines [17], the patient contributed to interpreting the qualitative findings for the meta-synthesis and meta-summary. Preliminary results were subsequently shared with the patient to ask whether the results aligned with their lived experience. Feedback highlighted commonalities between the findings and the patient’s perceptions, enhancing the credibility and authenticity of the analysis. Our iterative process ensured that the synthesis accurately captured and reflected the lived experiences and perspectives of movement in individuals with FM [8].

## Results

### Study selection

**Fig. 1:**
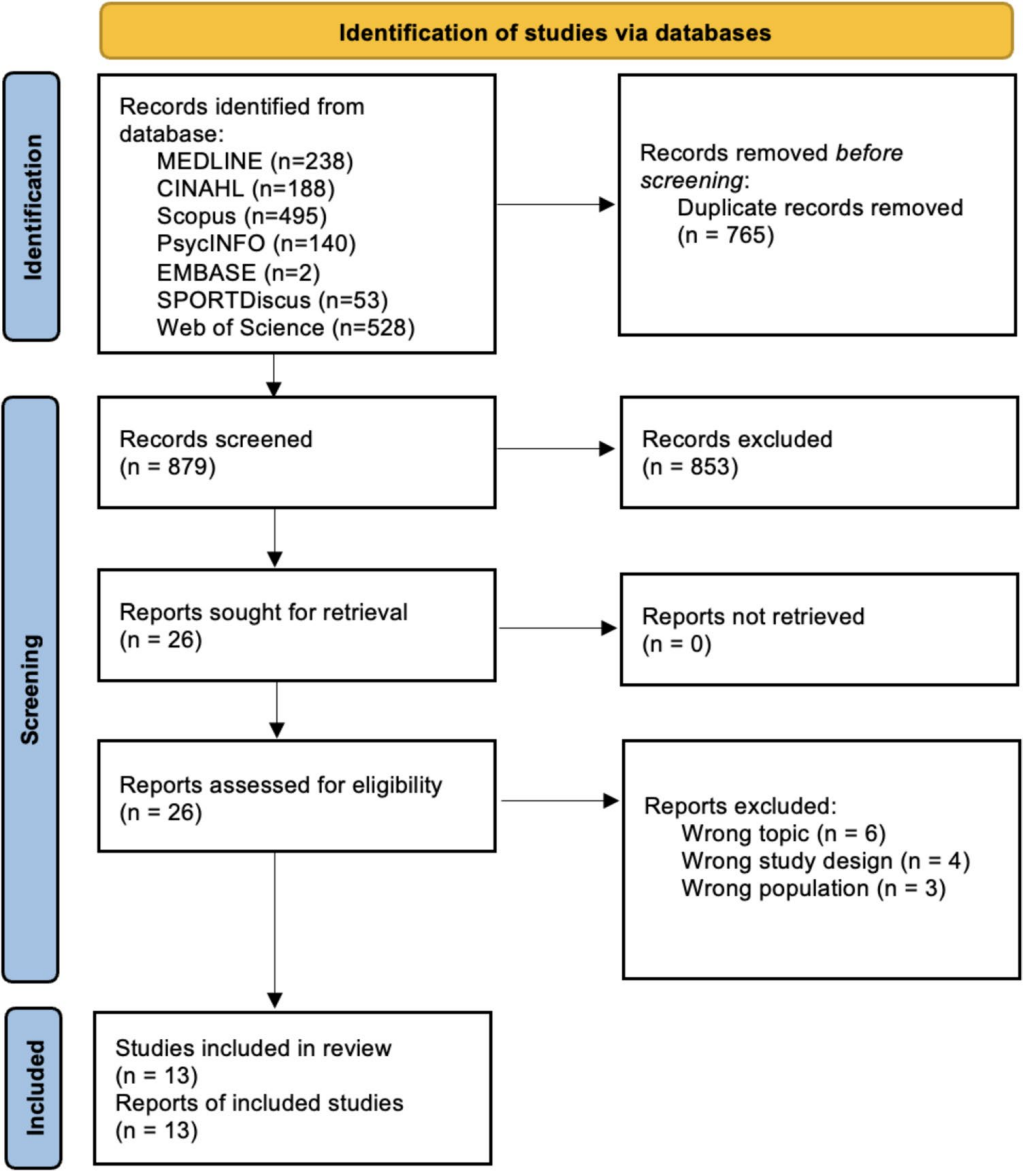
PRISMA flow diagram of the included studies

Out of 1587 articles obtained after the removal of the duplicates, 26 articles were identified with the title and abstract reading. After reading the 26 full texts, 13 were excluded for wrong topic (*n* = 6), wrong study design (*n* = 4), and wrong population (*n* = 3). Supplementary File 7 reported all details. Finally, 13 articles [4, 11, 29, 30, 37, 38, 41, 48, 52, 53, 55, 57, 60] were included in the systematic review (Fig. 1).

### Characteristics of the included studies

Twelve studies [4, 11, 29, 30, 37, 38, 41, 48, 52, 53, 55, 60] had a qualitative design, and one was a mixed-method study [57]. A total of 213 participants with FM (204 female) were included (Table 1). Participants’ ages ranged from 19 to 82 years. Three studies were conducted in Spain [4, 52, 53], two in the USA [30, 60], two in Sweden [29, 37], two in the UK [38, 48], one in Spain and USA [41], one in Brazil [11], one in Turkey [55], and one in Japan [57]. The included studies were categorized as conceptual/thematic description [4, 29, 30, 41, 48, 52, 53, 55, 57] and interpretive explanations [11, 37, 38, 60].

**Table 1:** Characteristics of the included studies

Study	Country	Aim	Participants	Data collection	Data analysis	Declared themes of patients’ experience
Beltran-Carrillo et al. (2013)	Spain	To provide an in-depth description and analysis of the perceived physical and psychosocial benefits of participation	*N*: 25 Age: between 38 and 82 years Female: 25	In-depth interviews and focus group analyzed	Combined strategies of “conventional” (inductive) and “directed” (deductive) content analysis	1. Perceived Physical Benefit Reducing muscular stiffness and pain; increasing vitality and physical function; avoiding inactivity and its disabling consequences 2. Perceived Psychological Benefit: an opportunity for social leisure time; feeling understood and believed by people with the same problem; receiving and giving affection, support and help; learning and developing a positive attitude for facing FM
Cavaliere et al. (2010)	Brazil	To identify FM patients’ perceptions of the relationship between physical exercise and health	*N*: 12 Age: between 21 and 61 years Female: 12	Semi-structured interviews	Theoretical- methodological framework of Social Representations	Physical dimension: pain relief; pain control; tolerating and coexisting with pain; resumption of activities of daily living; reduced fatigue; greater physical disposition; aesthetics Emotional dimension: increased self-esteem; reduced depression; greater well-being; improved emotional state Intellectual dimension: exercise good for health regardless of pain; adoption of a healthy lifestyle independent of disease; recognition of FM symptoms; awareness of missing exercise; recognition of limits; postural education; body awareness Social dimension: group interaction and motivation Spiritual dimension: improved state of mind
Larsson et al. (2019)	Sweden	To search for deeper knowledge of factors promoting physical activity in women with FM	*N*: 14 Age: between 38 and 65 y.o Sex: Female	Qualitative semi-structured in-depth individual interviews	Qualitative content analysis	A desire to be physically active: a need to be physically active; fear of getting worse; previous good experiences of physical activity Finding the proper level and creating proper conditions: need of adjustment; need of adequate knowledge and understanding Managing pain: accepting pain; balancing pain; prioritizing Getting it done: not giving up; exercise that feels good; support to get it done; accessibility; continuity
Lazaridou et al. [30]	USA	To: (1) understand the aspects of the experience that patients found most meaningful and beneficial, (2) clarify the relative importance of group and solo practice, and (3) gain insight into important logistical and practical considerations in implementing yoga practice into the daily life of individuals with FM	*N*: 15 Age: 50 ± 14.3 years Female: 15	Semi-structured interviews	Thematic analysis	Physical/body perceptual changes Practices affecting pain Emotional changes Practice motivators and barrier Group effect
Mannerkorpi et al. (2003)	Sweden	To study how patients with FM experienced physiotherapy group treatment comprising pool exercise and education	N: 19 Age: between 28 and 59 years Female: 19	Semi-structured interviews	Phenomenological approach	1. A positive experience of the body: experiencing relaxation; experiencing physical capacity; acknowledging limited capacity; changing the pattern of physical activity 2. Sharing experiences of living with FM; not being alone-receiving confirmation; sharing joy-distancing from illness 3. Creating new patterns of thinking and acting: calming down; creating a new relationship to body and self; creating a new relationship to social role; creating new patterns for managing pain
Mayana et al. (2021)	UK	To explore the perception of PA behavior and pain perception and the impact of psychosocial, contextual, and environmental factors on physical activity behavior and pain perception	*N*: 12 Age: between 20 and 70 years old Female: 10	Interviews	Critical realist ontology and epistemology	1. Lack of guidance in adapting suitable physical activity: positive perception of physical activity; fear of excruciating pain after physical activity; impact of physical activity intensity on pain; impact of weather conditions on physical activity; insufficient understanding of physical activity 2. Impact of fatigue on physical activity and pain: impact of physical fatigue and pacing; impact of mental fatigue on physical activity 3. Impact of treatment: impact of psychological intervention on acceptance and coping strategy; medication impacting negatively 4. Social impact: the impact of physical burden on work; impact of employer support; perceived work discrimination; impact of family support
Montesó-Curto et al. [41]	Spain and USA	To explore if men diagnosed with FMS in USA and Spain engaged in any type of physical activity or exercise, and if so, its frequency, intensity, and perceived effects from exercise	*N*: 17 (10 from Spain, 7 from US) Age: between 30 and 63 years Female: 0	Spain: Two focus group sessions USA: joint interview (*N* = 2), individual interviews (*N* = 5)	Qualitative content analysis	Understanding what constitutes physical activity or exercise Facilitating or discouraging the performance of physical exercise Effects of physical activity or exercise on psychological and social symptoms
[48]	UK	To explore the perceptions of fatigue and sleep dysfunction and exercise in people with FM	Number: 14 Age: > 18 years Female: 12	Focus group	Thematic content analysis	Lack of understanding: Sense of loss Impact of symptoms on the participants
Sanjuan-Sánchez et al. [52]	Spain	To describe the strategies and adaptations women with FM use to carry out basic, instrumental, and advanced activities of daily living	*N*: 25 Age: NR Female: 23	Interview	Thematic analysis	Basic ADLs: limitations experienced; movement and moving around; selfcare; strategies and adaptations; change of the activities; change of the posture Instrumental ADLs: limitations experienced; go shopping; housekeeping; cooking; cleaning; strategies and adaptations; investing more time; changing position; delegating; persisting; renouncing Advanced ADLs: limitations experienced; feeling exhausted at work; feeling stressed at work; not being capable; being forced to leave their jobs; strategies and adaptations; sitting; changing position; use of protective gear; non-stop work approach; avoiding overexertion
Sanz-Baños et al. [53]	Spain	To improve therapeutic interventions and increase adherence to walking as a foundation of interventions in patients suffering from FM	*N*: 46 Age: between 18 and 70 years Female: 46	Focus group	Qualitative content analysis	Behavioral beliefs: complaints about the behavior: physical, cognitive, and emotional discomfort; overload consequences; improved physical condition, symptoms, and health; self-esteem and well-being Normative beliefs: environment; family; health professionals; people with FM; myself Control beliefs: adherence to a fixed program; low self-efficacy; physical and emotional problems; daily responsibilities; environmental support; ideal circumstances for walking Psychosocial repercussion of living with FM: disease consequences; reassertation; other physical activity; association support
Sermenli et al. [55]	Turkey	To understand PA behaviors and beliefs in FM patients to address intervention needs	*N*: 10 Age: between 18 and 65 years Female: 10	Semi-structured interviews	Qualitative content analysis	Lack of knowledge: FITT (frequency, intensity, time, type of exercise) recommendations; definition and differences of the terms PA and exercise Person-centered approach: desire to take tailored advice Health benefits: lose weight; reduce the symptoms Past negative experiences: inappropriate exercise programs; unrealistic expectations Environmental barriers: location/accessibility; safety issues Personal barriers: lack of money; lack of time; lack of motivation Incorporate PA into daily life; set a specific time; break up into short session Constructive social dialogs: positive mindset; peer support; family support
Takai et al. (2022)	Japan	To design a 3-week inpatient exercise protocol and evaluate its effects through both quantitative scales and qualitative patient interviews	*N*: 12 Age: between 37 and 49 years Female: 12	Mixed-method	Thematic analysis	Reduction and localization of pain—from widespread, overwhelming pain to more localized, manageable sensations Discovery of bodily capability—surprise at tolerating moderate exercise; increased confidence Shift from fear-avoidance to active coping—movement perceived as alleviating rather than worsening pain Positive emotional transformation—emerging hope, life goals, and social re-engagement
Van Ravenstein (2014)	USA	To explore exercise barriers and strategies for women with FM and to formulate the basis for a practical and effective exercise intervention for this patient population by discovering the preferences and behaviors o f a group of women with FM	*N*: 6 Age: > 18 years Female: 6	Semi-structured interviews	Grounded Theory	Obtaining knowledge Develop strategies Overcome barriers Mantainance of PA

*ADL* activity of daily living, *N* number, *NR* not reported, *FM* fibromyalgia, *PA* physical activity

### Critical appraisal

Regarding the CASP assessment, items one through five and eight through ten were rated as “Yes” in all studies. Item six (researcher-participant relationship) was rated as “Yes” in four studies. Item seven (ethical issues) was rated as “Yes” in eight studies. Regarding the MMAT, one study [57] was assessed for screening questions and rated as “Yes.” Items one and three were rated as “Yes.” Items two and four were rated as “Can’t tell,” and item five was rated as “No.”

Detailed results of the quality appraisal using the CASP and MMAT are presented in Tables 2 and 3, respectively.

**Table 2:**
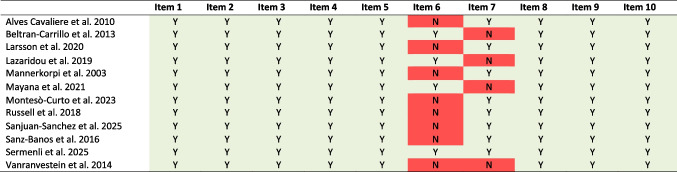
Critical Appraisal Skills Programme (CASP) results

*Y* yes, *N* no

Item 1: Was there a clear statement of the aims of the research?

Item 2: Is a qualitative methodology appropriate?

Item 3: Was the research design appropriate to address the aims of the research?

Item 4: Was the recruitment strategy appropriate to the aims of the research?

Item 5: Was the data collected in a way that addressed the research issue?

Item 6: Has the relationship between researcher and participants been adequately considered?

Item 7: Have ethical issues been taken into consideration?

Item 8: Was the data analysis sufficiently rigorous?

Item 9: Is there a clear statement of findings?

Item 10: How valuable is the research?

**Table 3:**
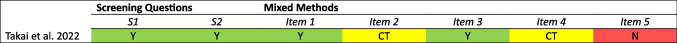
Mixed-Method Appraisal Tool (MMAT) results

*Y* yes, *CT* can’t tell, *N* no

S1. Are there clear research questions?

S2. Do the collected data allow to address the research questions?

Item 1. Is there an adequate rationale for using a mixed-methods design to address the research question?

Item 2. Are the different components of the study effectively integrated to answer the research question?

Item 3. Are the outputs of the integration of qualitative and quantitative components adequately interpreted?

Item 4. Are divergences and inconsistencies between quantitative and qualitative results adequately addressed?

Item 5. Do the different components of the study adhere to the quality criteria of each tradition of the methods involved?

### Meta-synthesis and meta-summary

**Fig. 2:**
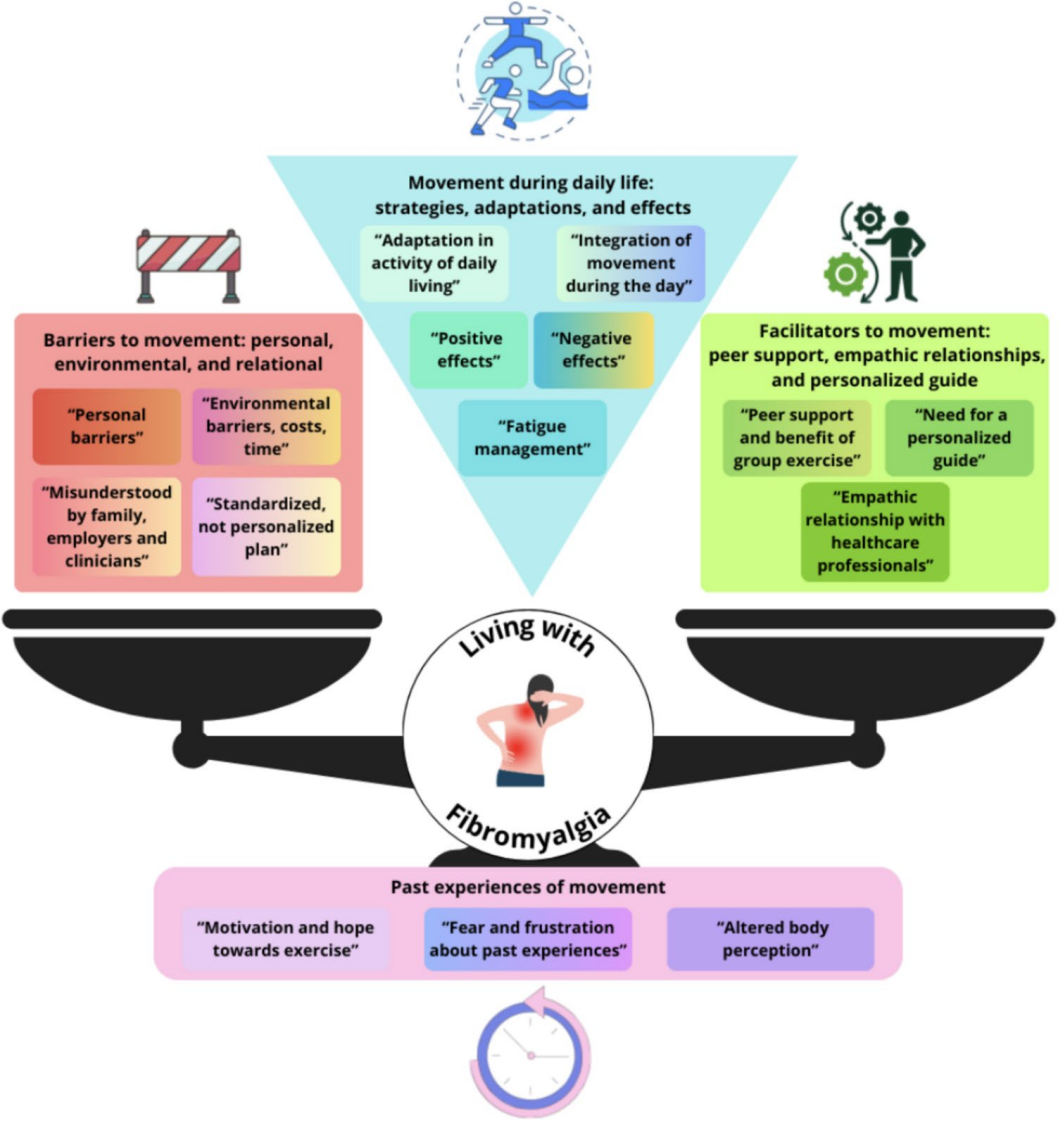
Four themes emerged from the systematic review: (1) past experiences of movement; (2) movement during daily life: strategies, adaptation, and effects; (3) barriers to movement: personal, environmental, relational; and (4) facilitators to movement*:* peer support, empathic relationship, and personalized guide

A total of 432 statements were extracted, analyzed, summarized, and grouped into 15 categories and four main themes (Fig. 2): (1) past experiences of movement; (2) movement during daily life: strategies, adaptation, and effects; (3) barriers to movement: personal, environmental, relational; and (4) facilitators to movement*:* peer support, empathic relationship, and personalized guide. The meta-summary highlighted that the categories with the highest inter-study frequency effect size were “Positive effects” (77%) and “Altered body perception” (69%), whereas the lowest was “Standardized, not personalized plan” (31%). For intra-study intensity effect size, the largest was reported by Sanz-Banos et al. [53] (93%) and Mayana et al. [38] (87%). The least frequent was reported by Cavaliere et al. [11] (13%) (Table 4).

**Table 4:** Meta-summary

Theme	Category	Studies													Interstudy frequency effect sizes (%)
		*Beltrán-Carrillo* et al*. (2013)*	*Cavaliere* et al*. (2010)*	*Larsson* et al*. (2020)*	*Lazaridou* et al*. (2019)*	*Mannerkorpi* et al*. (2003)*	*Mayana K.I* *(2021)*	*Montesó-Curto* et al*. (2023)*	*Russell* et al*. (2018)*	*Sanjuán-Sánchez* et al*. (2025)*	*Sanz-Banos* et al*. (2016)*	*Sermenli* et al*. (2025)*	*Takai* et al*. (2022)*	*VanRavenstein K (* *2014)*	
Past experiences of movement	Motivation and hope towards exercise	✓		✓	✓		✓	✓			✓	✓	✓	✓	69%
	Fear and frustration about past experiences			✓			✓	✓	✓	✓	✓	✓			54%
	Altered body perception			✓	✓	✓	✓	✓	✓	✓	✓		✓		69%
Movement during daily life: strategies, adaptations and effects	Adaptation in the activity of daily living					✓	✓	✓		✓	✓		✓		46%
	Fatigue management					✓	✓	✓	✓	✓	✓		✓	✓	62%
	Integration of movement during the day				✓			✓	✓		✓	✓		✓	46%
	Positive effects	✓	✓		✓	✓	✓	✓			✓	✓	✓	✓	77%
	Negative effects			✓			✓	✓	✓	✓	✓			✓	54%
Barriers to movement: personal, environmental, and relational	Personal barriers				✓		✓	✓	✓	✓	✓		✓	✓	62%
	Environmental barriers, costs, time			✓			✓			✓	✓	✓			38%
	Misunderstood by family, employers and clinicians						✓	✓	✓	✓	✓	✓			46%
	Standardized, not personalized plan						✓	✓	✓					✓	31%
Facilitators to movement: peer support, empathic relationship and personalized guide	Peer support and benefit of group exercise	✓	✓	✓	✓	✓	✓				✓	✓			62%
	Empathic relationship with healthcare professionals			✓	✓					✓	✓		✓		38%
	Need for a personalized guide			✓	✓	✓	✓	✓			✓	✓	✓		62%
**Intrastudy intensity effect sizes (%)**		20%	13%	53%	53%	40%	87%	80%	53%	60%	93%	53%	53%	47%	

Interstudy frequency effect size = (number of studies containing a theme/total number of studies) × 100

Intrastudy intensity effect size = (number of findings in the study/total number of themes) × 100

### Theme 1: Past experiences of movement

The first theme explained how participants experienced movement in the past. It was derived from “Motivation and hope towards exercise,” “Fear and frustration about past experiences,” and “Altered body perception” categories.

#### “Motivation and hope towards exercise”

Some patients were aware of the benefits that physical activity can bring, and especially how much an inactive lifestyle can worsen their condition. They therefore appeared particularly motivated to participate in exercise programs and determined to maintain an active lifestyle [4, 29, 30, 38, 41, 53, 55, 57, 60]. People described moving their bodies as a protective, hopeful act that prevents decline and sustains everyday life [4]. Motivation was often visceral, an intrinsic need to be active, coupled with a fear of inactivity, and movement reliably felt better than staying sedentary [29]. Hope was nourished by longing to keep trying, a mental–physical “memory” of activity, and the conviction that there was always something one could do, even when walking is hard ^29,53^. Through practice, self-efficacy grew, and concrete preferences emerged (e.g., choosing yoga at home) ^30^.

#### “Fear and frustration about past experiences”

People also described substantial barriers and apprehensions toward exercise: generic gyms or standard classes often did not work for them despite repeated trials [29]; negative experiences, like swimming that triggers spasms, led to avoidance [38]; forcing activity frequently worsens pain during and especially after, with little benefit for persistent fatigue and episodes of breathlessness and fluctuating, “classic FM” symptoms [41]. Everyday tasks illustrated the fragility of function: changing a lightbulb can provoke day-long pain; bending, kneeling, showering, drying hair, or even doing the dishes becomes difficult, making “exercise” itself feel like a “scary word” [41, 48, 52].

#### “Altered body perception”

Participants described a renegotiated relationship with their bodies, accepting persistent pain, diverting attention, and making conscious “cost–benefit” trade-offs [29]. Structured programs, especially yoga, helped some recalibrate limits, rebuild confidence, and “feel” the body in healing ways [30]. Yet altered perception also included frustration with reduced strength and difficulty accepting a changed body and a sense of fighting against progressive decline to avoid becoming housebound [37].

### Theme 2: Movement during daily life: strategies, adaptations, and effects

The second theme focused on how people with FM manage and integrate movement into their daily lives, what adaptations they make to best perform their daily activities, and what physical and psychological effects this has. It is derived from “Adaptation in activity of daily living,” “Fatigue management,” “Integration of movement during the day,” “Positive effect,” and “Negative effects” categories.

#### “Adaptation in activity of daily living”

Participants described pragmatic and often creative adjustments to preserve their autonomy.

They renegotiated roles by asking for help with heavy tasks at work—something they “never did before”—and, when needed, reducing or leaving employment, noting that even seated roles can feel demanding with FM [37, 38]. Energy-conservation strategies included planned recovery after basic hygiene (e.g., needing 15 min after a shower), splitting chores into stages (peeling potatoes in batches), and adopting appearance shortcuts (keeping a beard to avoid the effort of shaving) [41]. Home and self-care were adapted with long-handled aids, towels used as reach extenders, grab bars, shower-tray conversions and anti-slip devices, choosing showers over baths when insecure, avoiding carpets to minimize cleaning, and prioritizing supportive footwear [52, 53].

#### “Fatigue management”

Participants described pacing as a deliberate, values-based practice: they deprioritize nonessential chores, learn to say “no,” and accept doing tasks tomorrow—or only partly—without guilt, assessing each morning what feels feasible [37]. Fatigue was managed by stopping at the first signs of achiness, taking restorative showers and rests, and using medication or quiet time when needed [38]. They seek the “middle path” between inactivity (stiffness) and overexertion (days bed-bound), heed the “good-day trap” by cutting back even when energy is high, and calibrate effort to prevent flare-ups [38, 41].

#### “Integration of movement during the day”

Participants embedded short, routine-friendly bouts of movement into everyday contexts: a half-hour of morning yoga boosts alertness, and brief breathing exercises are used “on the go” (e.g., in traffic), with a personal target of practicing at least every other day [30]. Many relied on incidental activity rather than formal programs—climbing household stairs 15–17 times daily, performing stretching 5–10 times a day despite transient pain relief, and folding in yard work, shopping, and driving—while voicing a persistent longing to keep exercising and the importance of secure footing for confidence and safety [41, 48, 52].

#### “Positive effects”

Participants reported multi-level gains from movement. Even small amounts of exercise brought pain relief and made daily life more manageable, alongside an increase in energy, mobility, and agility [4, 37]. Learning better breathing and body mechanics enhanced functional confidence and self-esteem, while practices like yoga increased body awareness, got the body “moving again,” built stamina, deepened sleep, and left people feeling lighter and more capable (“no elephant on my shoulder”) [30, 37].

#### “Negative effects”

Participants described activity-triggered exacerbations that could feel like a “hangover,” with a transient high followed by being “completely knocked out” the next day [29]. Seemingly reasonable efforts—yoga, dance classes, or extending a planned session—could precipitate intense pain and exhaustion lasting days (“wrecked… everything hurts,” “extremely tired,” and “bed-bound for days”), prompting hot showers, rest, and analgesics and sometimes deterring future attempts [38, 41]. Pushing beyond one’s limit was linked with “brutal” joint pain, cramps, and prolonged sickness; even brief outings could necessitate a day in bed, and some reported feeling worse “after the minimum exercise” [41, 48].

### Theme 3: Barriers to movement: personal, environmental, and relational

The third theme highlights the participants’ barriers to performing physical exercise. It derives from “Personal barriers,” “Environmental barriers, costs, time,” “Misunderstood by family, employers and clinicians,” and “Standardized, not personalized plan” categories.

#### “Personal barriers”

Participants depicted a dense web of impediments, including pain, fatigue, sleep, mood, medication effects, and role disruptions. Pain flared with specific positions or loads (e.g., kneeling), sometimes making even minimal movement “impossible,” while pervasive, non-restorative fatigue abruptly halted activity, demanded long naps, blunted concentration, and undermined daytime capacity [30, 38]. Sleep was fragmented, fueling guilt about parenting and reliance on partners for basic chores like carrying laundry, climbing stairs, or cooking [52].

#### “Environmental barriers, costs, time”

Participants described a web of contextual constraints that derailed exercise despite good intentions. Competing roles, paid work, childcare, elder care, and housework compressed time and energy, making adherence fragile and prompting late starts or missed sessions [29, 52, 53]. Financial insecurity and costs further limited participation: unemployment, bills, and high class fees made regular programs “hit and miss,” with lockdown disruptions adding instability [38, 52, 55]. Participants imagined retirement as a window for self-care [37]. Even strong intentions (“I routinely want to exercise every day”) faltered when routines could not accommodate activity; by day’s end, fatigue and competing duties prevailed, leading some to quit despite recognizing exercise’s value [55].

#### “Misunderstood by family, employers and clinicians”

Participants recounted a pervasive sense of invalidation and disbelief across care, work, and home. Some perceived outright skepticism from health personnel (“they don’t believe you”), which eroded trust after years of escalating prescriptions and fueled a resolve to resist further dose increases [38, 52]. At work, misunderstanding translated into discrimination and procedural injustice: health-screening rejections, premature dismissal during medical retirement processes, pressure to return because one “looks fine,” and fear that disclosing FM will jeopardize employment; inadequate guidance after injuries compounded losses [38, 52]. Within families, limited practical help and the invisibility of symptoms fostered judgements of laziness or difficulty; relatives “can’t comprehend” exhaustion after small efforts, and condescending questions (e.g., “Is it good for you to exercise?”) stung.

#### “Standardized, not personalized plan”

Participants reported non-individualized, poorly implemented guidance that often worsens symptoms rather than supporting sustainable activity: instructors and trainers appeared unfamiliar with chronic pain, prescribing routines that ignore joint-specific limitations (e.g., “50 squats” despite knee pain), while classes felt generic and not condition-aware [38]. Clinical advice was frequently weight-centric or equipment-oriented without concrete implementation, progression, or FM-specific strategies [41]. Across accounts, participants called for FM-tailored pacing and education that integrates symptom management with carefully dosed activity, “start low and go slow,” instead of one-size-fits-all exercise guidelines [38, 41, 60].

### Theme 4: Facilitators to movement: peer support, empathic relationship, and personalized guide

The final theme highlighted the conditions that facilitate participants’ maintenance of an active lifestyle, supported through movement. It derives from “Peer support and benefit of group exercise,” “Empathic relationship with healthcare professionals,” and “Need for a personalized guide.”

#### “Peer support and benefit of group exercise”

Participants portrayed group-based activity as a psychological lifeline: even after a crowded commute, being with peers “in the gym” lifts mood and counters loneliness [11], while shared sessions distract from pain (“your mind is on something else”) and feel therapeutic in themselves [4]. Peer spaces validated experience, offer practical advice, and even nudge identity shifts toward brighter self-presentation, reinforcing that the group “is great for us as a therapy” [4]. Feeling “noticed” on arrival mattered [29], and condition-specific communities normalized symptoms, dispelled catastrophic fears (“am I going to end up in a wheelchair?”), and allowed light-hearted coping [37]. Walking companions and patient associations reduced isolation, provided empathy that outsiders often lack, and motivated continued activity; members described “supporting each other a lot” and mentoring newcomers, while also curating their social milieu, seeking communicative, solution-oriented peers and avoiding persistently pessimistic groups that “bring people down” [53, 55].

#### “Empathic relationship with healthcare professionals”

Participants highlighted how sensitive, individualized care unlocks engagement and hope.

They valued clinicians who titrate load, avoid coercion, and validate limits (“so patients don’t “go home and feel sad”) and instructors whose warmth fostered commitment and adherence [29, 30]. Trust grew when doctors “work around” system gaps (e.g., recognizing osteoarthritis to authorize sick leave when FM isn’t acknowledged) and when primary care and psychology offered steady encouragement to move within safe bounds (e.g., brief walks) [52, 53]. In rehabilitation, empathic physiotherapists balanced encouragement (“try one more time”) with explicit permission not to overdo, provided soothing hands-on care, ensured continuity across therapists, and proactively noticed worries, reducing anxiety and affirming a right to “live at my own pace” ^57^.

#### “Need for a personalized guide”

Participants asked for a disease-informed coach who is available to consult, adjusts load slowly, and tweaks exercises without constant overhauls (“someone to support me… and help me progress slowly”) [29]. Education and skills training helped break the pain–inactivity spiral and foster acceptance of “doing what I can” within new limits [38]. Therapist presence was crucial: proper footwear and individualized guidance revealed unexpected capacity; therapists identified tense regions, taught how to loosen them, and coached cognitive reframing (“I must not indulge myself”) toward listening to one’s condition and balancing effort and rest [57]. Graded assistance (staying nearby at the start, accompanying resistance, explaining clearly, and encouraging at the right moments) built confidence and continuity [57].

### Confidence assessment

Overall, the review findings were assessed with moderate confidence. Across themes, the main methodological concerns related to limited reporting of researcher reflexivity and the researcher-participant relationship (CASP item 6 was rated “No” in 8/12 qualitative studies) and, less frequently, incomplete reporting of ethical considerations (CASP item 7 was rated “No” in 4/12). Adequacy and coherence were judged evidentially rather than by counts alone: adequacy reflected the volume and richness of data supporting each theme, and coherence reflected the clarity and consistency of the underlying pattern within and across studies. Theme-specific CERQual judgements, including the key limitations that informed each component and brief examples of the supporting data, are reported in Table 5 (with extended supporting material in Supplementary File 3).

**Table 5:** GRADE-CERQual summary of findings

	Summarized review finding	Methodological limitations	Coherence	Adequacy	Relevance	GRADE-CERQual assessment of confidence	References
1	Past experiences of movement	*Minor concerns* **Explanation:** Key limitations involved limited reporting of reflexivity and researcher-participant relationships (CASP item 6) and, in a minority, ethics procedures (CASP item 7). These limitations were unlikely to materially alter the meaning of this theme because accounts largely described participants’ own past experiences of movement	*Minor concerns* **Explanation:** A coherent pattern emerged across 12/13 studies: movement was portrayed as simultaneously hopeful/protective and risky, with fear and frustration linked to symptom exacerbations. Variation reflected individual thresholds and day-to-day variability rather than conflicting interpretations	*Minor concerns* **Explanation:** The theme drew on 12 studies and included moderately rich descriptions of motivation, negative experiences, and altered body perception. Supporting data included accounts of repeated attempts to remain active, avoidance after pain/fatigue flares, and rebuilding confidence through graded or mind–body practices	*Moderate concerns* **Explanation:** Evidence predominantly reflected women and high-income settings, although contributing studies spanned Europe, North America, Asia, and South America	*Moderate confidence* **Explanation:** Minor concerns regarding methodological limitations, Minor concerns regarding coherence, Minor concerns regarding adequacy, and Moderate concerns regarding relevance	Beltrán-Carrillo et al. [4],Larsson et al. (20,200; Lazaridou et al. [30],Mannerkorpi and Gard [37],Mayana; Montesó-Curto et al. [41],Russell et al. [48],Sanjuan-Sánchez et al. [52],Sanz-Baños et al. [53],Sermenli et al. [55],Takai (2022); VanRavenstein [60]
2	Movement during daily life: strategies, adaptations and effects	*Minor concerns* **Explanation:** As above, the main limitations related to limited reflexivity (CASP item 6) and occasional incomplete ethics reporting (CASP item 7). These issues were unlikely to change the overall pattern of day-to-day self-management strategies reported	*Minor concerns* **Explanation:** Findings were consistent across 9 studies in showing how participants balanced under-activity (stiffness) and over-activity (flares) through pacing and adaptation. Reports of benefits (e.g., improved energy/function) coexisted with delayed symptom exacerbations without undermining the overall pattern	*Minor concerns* **Explanation:** The theme was supported by 9 studies with concrete, practice-relevant descriptions of adaptations, such as splitting chores, planning rests, using aids, and embedding short bouts of movement (e.g., yoga or breathing) into routines, alongside descriptions of both positive and negative effects	*Minor concerns* **Explanation:** Studies covered multiple countries and settings broadly aligned with the review question on exercise and everyday movement	*Moderate confidence* **Explanation:** Minor concerns regarding methodological limitations, Minor concerns regarding coherence, Minor concerns regarding adequacy, and Minor concerns regarding relevance	Beltrán-Carrillo et al. [4],Cavaliere (2010); Larsson et al. [29],Lazaridou et al. [30],Mannerkorpi and Gard [37],Mayana; Montesó-Curto et al. [41],Russell et al. [48],Sanjuan-Sánchez et al. [52],Sanz-Baños et al. [53],Sermenli et al. [55],Takai (2022); VanRavenstein [60]
3	Barriers to movement: personal, environmental, relational	*Minor concerns* **Explanation:** The appraisal highlighted limited reflexivity (CASP item 6) and, in some studies, incomplete ethics reporting (CASP item 7). These limitations were unlikely to change the identification of commonly reported barriers	*Minor concerns* **Explanation:** Barriers converged across 9 studies, including symptom unpredictability and fatigue, competing roles and limited time, costs and access issues, environmental constraints, invalidation from others, and non-tailored advice. The pattern was stable across contexts	*Minor concerns* **Explanation:** The theme drew on 9 studies and included specific examples of barriers at home, work, and healthcare, such as financial constraints, safety/access issues, perceived disbelief by clinicians or employers, and experiences with generic, non-individualized exercise guidance	*Moderate concerns* **Explanation:** Evidence reflected a predominance of women and a limited range of cultural and healthcare contexts, which may constrain transferability	*Moderate confidence* **Explanation:** Minor concerns regarding methodological limitations, Minor concerns regarding coherence, Minor concerns regarding adequacy, and Moderate concerns regarding relevance	Larsson et al. [29],Mayana; Montesó-Curto et al. [41],Russell et al. [48],Sanjuan-Sánchez et al. [52],Sanz-Baños et al. [53],Sermenli et al. [55],Takai (2022); VanRavenstein [60]
4	Facilitators to movement: peer support, empathic relationship and personalized guide	*Moderate concerns* **Explanation:** Limited reporting of reflexivity and researcher-participant relationships (CASP item 6) was particularly pertinent for this relational theme. In addition, several contributing studies were conducted in structured program or rehabilitation contexts, which may have shaped accounts of support and guidance	*Minor concerns* **Explanation:** Facilitators were described consistently across studies, with convergent accounts of peer support, empathic and validating healthcare relationships, and the value of personalized progression and guidance	*Minor concerns* **Explanation:** The theme drew on all 13 studies and provided moderately rich descriptions of how supportive social contexts and tailored guidance enabled sustained activity, including examples such as group-based validation, reassurance about safe limits, and gradual load adjustment	*Minor concerns* **Explanation:** Studies spanned multiple countries and settings relevant to movement and exercise experiences in fibromyalgia	*Moderate confidence* **Explanation:** Moderate concerns regarding methodological limitations, Minor concerns regarding coherence, Minor concerns regarding adequacy, and Minor concerns regarding relevance	Beltrán-Carrillo et al. [4],Cavaliere (2010); Larsson et al. [29],Lazaridou et al. [30],Mannerkorpi and Gard [37],Mayana; Montesó-Curto et al. [41],Sanjuan-Sánchez et al. [52],Sanz-Baños et al. [53],Sermenli et al. [55],Takai (2022); VanRavenstein [60]

*CASP* Critical Appraisal Skills Programme

## Discussion

This review synthesizes how people with FM interpret, attempt, and sustain movement. Four interlocking themes emerged: (1) past experiences of movement with FM; (2) movement during daily life with FM: strategies, adaptation, and effects; (3) barriers to movement: personal, environmental, relational; and (4) facilitators to movement*:* peer support, empathic relationship, and personalized guide. Across studies, participants’ accounts repeatedly referred to moments of starting, pausing, and resuming activity; in this review, these moments are interpreted as “decision points” that help organize how the identified themes are negotiated, rather than as a formal synthesized model. The meta-summary highlighted that the categories with the highest inter-study frequency effect size were “Positive effects” (77%) and “Altered body perception” (69%), whereas the lowest was “Standardized, not personalized plans” (31%). For intra-study intensity effect size, the highest values were observed in Sanz-Banos et al. [53](93%) and Mayana et al. [38] (87%), while the lowest was reported by Cavaliere et al. [11] (13%). Although these findings deepen the understanding of the experiences of people with FM [4, 11, 29, 30, 37, 38, 41, 48, 52, 53, 55, 57, 60], the GRADE-CERQual assessment indicated moderate confidence that the meta-synthesis reflects FM patients’ experiences of movement and the findings should be interpreted with caution.

### Comparison with evidence

For the first theme, participants’ past experiences reflect a persistent ambivalence: movement is remembered as helpful and hazardous, including episodes of “crashes” after minor efforts mold caution, self-efficacy, and altered body perception [4, 29, 30, 37, 38, 41, 48, 52, 53, 55, 57, 60]. This pattern further refines prior illness-experience meta-syntheses by linking legitimacy struggles directly to decisions about pacing, stopping, and re-entry after setbacks in relation to the oscillation between “struggling,” “adapting,” and “giving up” [56]. It also extends meta-ethnographic accounts of protracted diagnostic uncertainty, where skepticism and mixed relief cultivate vigilant body-listening, by illustrating why explicit permission to stop early and symptom-contingent dosing are perceived as rational safeguards rather than reluctance to engage [39]. Stigma colors these histories further, with defensive withdrawal reinforced when efforts were dismissed or pathologized in the past [18].

In the second theme, day-to-day movement is negotiated through practical tactics (energy budgeting, micro-bouts, pre-emptive rests, and translation of domestic/caregiving tasks into “dose”) that participants already use but want clinicians to formalize [4, 11, 29, 30, 37, 38, 41, 48, 52, 53, 55, 57, 60]. This FM-specific detailing complements qualitative syntheses of self-management in chronic widespread pain by specifying how empowerment and flexible delivery are enacted at home [26]. Reports of perceived benefits (mood, sleep, and capability) co-exist with delayed payback, aligning with quantitative trends (high dropout in exercise trials and dose–response evidence advocating “start low, progress slow”) and explaining requests for explicit pacing and early warning “stop rules” [6, 35, 46, 59].

Within the third theme, personal barriers include symptom volatility, fear of post-exertional worsening, and uncertainty about safe thresholds; environmental barriers span cost, access, and weather; relational barriers center on invalidation in healthcare, family, and work [29, 30, 38, 41, 48, 52, 53, 55, 57, 60]. The current synthesis brings together such layers, mapping closely to primary-care evidence of bidirectional misunderstanding and time pressure, to stigma meta-synthesis findings, and to cross-condition analyses that identify pain burden, comorbidity, limited benefit knowledge, and time constraints as dominant impediments [7, 31, 39]. Its contribution is to show how such domains converge around moments in which participants describe deciding whether to start, pause, or resume activity, which we interpret as key “decision points” in the negotiation of everyday movement.

The fourth theme suggests that participants framed these facilitators not as optional add-ons, but as enabling conditions shaping how movement is experienced and negotiated. Peer contexts were described as normalizing variability and providing gentle accountability; empathic, continuous clinician relationships were perceived as being associated with a shift in the meaning of movement through validation and negotiated micro-progressions; and a personalized guide was described as helping translate symptoms into concrete rules for titration and re-entry [4, 11, 14, 29, 30, 37, 38, 41, 52, 53, 55, 57, 60]. These findings align with primary-care and treatment-experience syntheses where negotiated micro-progressions and combined, individualized approaches are preferred [7, 16, 39] and with self-management reviews emphasizing flexible formats and shared problem-solving [31]. When read alongside behavior-change mapping in chronic pain [31], the present review adds FM-specific levers—altered body perception, post-exertional cycles, and the “banking” of incidental load, helping explain why peer support and clinician continuity are perceived as enabling conditions for safe, sustained engagement rather than optional embellishments [18].

### Implication for clinical practice and research

Ambivalence has emerged as a rational product of past experiences, and care may benefit from explicitly recognizing this ambivalence [39, 56]. Clinicians should invite a brief “activity biography” that may surface memories of gains and crashes, the meanings attached to movement, and episodes of delegitimization. This opens space to validate body-listening and to co-author clear “permission to pause” without framing it as failure [13, 27, 29]. The studies identified barriers that call for attention to context: minimize sensory and organizational burden around sessions, align team messages to avoid mixed cues, and provide simple, shareable summaries that help families and employers understand fluctuating function [7, 27]. Participants favored peer support, continuity with an empathic clinician, and the presence of a personalized guide as facilitators, which were described as enabling conditions: groups that normalize variability were perceived as providing soft accountability, while attuned clinicians were perceived as helping reframe movement from threat to tool through validation of limits and negotiated exposure [48, 52, 55].

Future qualitative studies should stay close to the lived experience, charting the negotiations around decisions about starting, pausing, and re-entering activity over time. Longitudinal designs featuring diaries, brief follow-up interviews, or go-along observations capture the lagged consequences of everyday efforts and small adjustments made after setbacks [2, 23, 28]. Participatory and co-design approaches are apt for developing the “personalized guide,” family-facing summaries, and language that legitimizes pacing with iterative feedback from people with FM [3, 61]. Comparing services and cultures will clarify how environmental and relational barriers shape decision points; focused inquiries into peer contexts will unpack mechanisms by which groups reduce fear and sustain engagement [18, 26]. Purposeful sampling of underrepresented voices, including men, people with multimorbidity, and lower socioeconomic groups, alongside thick descriptions of context, will improve transferability and help specify when and for whom these qualitative insights hold.

### Strengths and limitations

This is the first qualitative systematic review to integrate patients’ experiences and perceptions of movement in patients with FM, undertaken with a transparent, prospectively registered protocol. The synthesis combined meta-synthesis and meta-summary per Sandelowski and Barroso and quantified inter-study frequency and intra-study intensity effect sizes, giving additional interpretability beyond purely narrative aggregation. Finally, interpretation was continuously peer-debriefed within a multidisciplinary team with experience in FM and qualitative synthesis, enhancing credibility and trustworthiness.

Despite the breadth of sources, several findings carried moderate concerns about relevance because included studies originated from a limited spread of countries/continents, which may constrain transferability across diverse health-system contexts. In addition, only a subset of available bibliographic databases was searched, so some relevant studies may not have been identified. This review did not exclude lower-quality studies a priori. Although this inclusive stand can minimize bias from selective omission, it may lead to dilution of overall certainty, although CASP and CERQual procedures were applied to temper inferences. Several synthesized domains have ultimately received moderate confidence ratings, underscoring the need for cautious application in settings dissimilar to those studied.

## Conclusion

This qualitative synthesis underscores that for people with FM movement is simultaneously valued and feared. Activity is perceived as a path to relief, capability, and social connection, yet it is also associated with delayed flares, exhaustion, and loss of control. Across studies, this ambivalence is not a contradiction but a rational response to unpredictable symptoms, delegitimizing encounters, and environments that make sustained engagement difficult. The review shows that explicit pacing and “crash-prevention” strategies were consistently perceived by participants as making movement more manageable when delivery was flexible. Empathic clinicians, peer support, and continuity of guidance were described as enabling conditions that helped reframe movement from threat to tool and supported confidence and legitimacy of variability.

## Supplementary Information

Below is the link to the electronic supplementary material.

ESM 1 Supplementary Material 1 (DOCX 15.3 KB)

ESM 2 Supplementary Material 2 (DOCX 29.9 KB)

ESM 3 Supplementary Material 3 (DOCX 17.2 KB)

ESM 4 Supplementary Material 4 (DOCX 19.6 KB)

ESM 5 Supplementary Material 5 (DOCX 87.0 KB)

ESM 6 Supplementary Material 6 (DOCX 21.9 KB)

ESM 7 Supplementary Material 7 (DOCX 17.1 KB)

## References

[1] Alciati A, Nucera V, Masala I et al (2021) One year in review 2021: fibromyalgia. Clin Exp Rheumatol 39:3–12. 10.55563/clinexprheumatol/gz4i3i 34001307 10.55563/clinexprheumatol/gz4i3i

[2] Bartlett R, Milligan C (2015) What is diary method? Bloomsbury Academic. 10.5040/9781472572578

[3] Bate P, Robert G (2006) Experience-based design: from redesigning the system around the patient to co-designing services with the patient. Qual Saf Healthc 15(5):307–310. 10.1136/qshc.2005.016527 10.1136/qshc.2005.016527 PMC2565809 17074863

[4] Beltrán-Carrillo VJ, Tortosa-Martínez J, Jennings G, Sánchez ES (2013) Contributions of a group-based exercise program for coping with fibromyalgia: a qualitative study giving voice to female patients. Women Health 53(6):612–629. 10.1080/03630242.2013.819399 23937732 10.1080/03630242.2013.819399

[5] Bidonde J, Busch AJ, Schachter CL, Overend TJ, Kim SY, Góes SM, Boden C, Foulds HJ (2017) Aerobic exercise training for adults with fibromyalgia. Cochrane Database Syst Rev 6(6):CD012700. 10.1002/14651858.CD012700 28636204 10.1002/14651858.CD012700 PMC6481524

[6] Busch AJ, Webber SC, Brachaniec M, Bidonde J, Bello-Haas VD, Danyliw AD, Overend TJ, Richards RS, Sawant A, Schachter CL (2011) Exercise therapy for fibromyalgia. Curr Pain Headache Rep 15(5):358–367. 10.1007/s11916-011-0214-2 21725900 10.1007/s11916-011-0214-2 PMC3165132

[7] Byrne A, Jones K, Backhouse M, Rose F, Moatt E, van der Feltz-Cornelis C (2023) Patient and primary care practitioners’ perspectives on consultations for fibromyalgia: a qualitative evidence synthesis. Prim Health Care Res Dev 24:e58. 10.1017/S1463423623000506 37750736 10.1017/S1463423623000506 PMC10540196

[8] Carcary M (2009) The research audit trial – enhancing trustworthiness in qualitative inquiry. Electron J Bus Res Methods 7(1):11–24

[9] Casanova-Rodríguez D, Ranchal-Sánchez A, Rodríguez RB, Jurado-Castro JM (2025) Aerobic exercise prescription for pain reduction in fibromyalgia: a systematic review and meta-analysis. Eur J Pain 29(2):e4783. 10.1002/ejp.4783 39805734 10.1002/ejp.4783 PMC11730678

[10] CASP (2018) Critical Appraisal Skills Programme (CASP) Qualitative Research Checklist. Available at: https://casp-uk.net/casp-tools-checklists/

[11] Cavaliere MLA, de Abreu Souza JM, de Oliveira Barbosa JS (2010) Representations of the relationship between physical exercise and health for patients with fibromyalgia. P. 20(4):1325

[12] Chinn S, Caldwell W, Gritsenko K (2016) Fibromyalgia pathogenesis and treatment options update. Curr Pain Headache Rep 20(4):25 26922414 10.1007/s11916-016-0556-x

[13] Cioeta M, Youssef S, Brindisino F, Venturin D, Pichero R, Giovannico G, Pournajaf S, Goffredo M, Caselli S, Pellicciari L (2025) Cross-cultural adaptation and psychometric properties of the Italian version of the Patient-Specific Functional Scale (PSFS) in subjects with shoulder pain. Disabil Rehabil 47(2):512–518. 10.1080/09638288.2024.2342495 38700257 10.1080/09638288.2024.2342495

[14] Ciolan F, Bertoni G, Crestani M, Falsiroli Maistrello L, Coppola I, Rossettini G, Battista S (2025) Perceived factors influencing the success of pain neuroscience education in chronic musculoskeletal pain: a meta-synthesis of qualitative studies. Disabil Rehabil 47(10):2459–2474. 10.1080/09638288.2024.2398141 39225055 10.1080/09638288.2024.2398141

[15] Clauw DJ (2014) Fibromyalgia: a clinical review. JAMA 311(15):1547–1555 24737367 10.1001/jama.2014.3266

[16] Climent-Sanz C, Hamilton KR, Martínez-Navarro O, Briones-Vozmediano E, Gracia-Lasheras M, Fernández-Lago H, Valenzuela-Pascual F, Finan PH (2024) Fibromyalgia pain management effectiveness from the patient perspective: a qualitative evidence synthesis. Disabil Rehabil 46(20):4595–4610. 10.1080/09638288.2023.2280057 37965900 10.1080/09638288.2023.2280057 PMC11093884

[17] Cochrane. Patient and public. Available at: https://www.cochrane.it/it/partecipa/cittadini-pazienti

[18] Colombo B, Zanella E, Galazzi A, Arcadi P (2025) The experience of stigma in people affected by fibromyalgia: a metasynthesis. J Adv Nurs 81(10):6317–6332. 10.1111/jan.16773 39835578 10.1111/jan.16773 PMC12460976

[19] Colvin CJ, Garside R, Wainwright M et al (2018) Applying GRADE-CERQual to qualitative evidence synthesis findings—paper 4: how to assess coherence. Implement Sci13(13). 10.1186/s13012-017-0691-8 10.1186/s13012-017-0691-8 PMC5791039 29384081

[20] Cooke A, Smith D, Booth A (2012) Beyond PICO: the SPIDER tool for qualitative evidence syn-thesis. Qual Health Res. 10.1177/1049732312452938 22829486 10.1177/1049732312452938

[21] Crestani M, Cook C, Ceccarelli E, Delladio S, Palese A, Turolla A, Maselli F, Mourad F, Gandolfi M, Rossettini G (2025) “I’m not the same as i was before”: a qualitative evidence synthesis exploring the experiences and perceptions of patients living with whiplash-associated disorders. J Orthop Sports Phys Ther 55(9):1–19. 10.2519/jospt.2025.13156 40879621 10.2519/jospt.2025.13156

[22] Crestani M, Cook C, Leuci C, Carletto L, Garzonio F, Palese A, Chester R, De Baets L, Gandolfi M, Rossettini G, Brindisino F (2025) “I cannot recognize my body”: experiences and perceptions of patients living with frozen shoulder: a qualitative systematic review with meta-synthesis and meta-summary. J Orthop Sports Phys Ther 55(9):1–16. 10.2519/jospt.2025.13432 40875588 10.2519/jospt.2025.13432

[23] Garcia-Palacios A, Herrero R, Belmonte MA, Castilla D, Guixeres J, Molinari G, Baños RM (2014) Ecological momentary assessment for chronic pain in fibromyalgia using a smartphone: a randomized crossover study. Eur J Pain 18(6):862–872. 10.1002/j.1532-2149.2013.00425.x 24921074 10.1002/j.1532-2149.2013.00425.x

[24] Glenton C, Carlsen B, Lewin S et al (2018) Applying GRADE-CERQual to qualitative evidence synthesis findings—paper 5: how to assess adequacy of data. Implement Sci 13(14). 10.1186/s13012-017-0692-7 10.1186/s13012-017-0692-7 PMC5791045 29384077

[25] Hong QN, Fàbregues S, Bartlett G, Boardman F, Cargo M, Dagenais P, Gagnon M-P, Griffiths F, Nicolau B, O’Cathain A, Rousseau M-C, Vedel I, Pluye P (2018) The Mixed Methods Appraisal Tool (MMAT) version 2018 for information professionals and researchers. Educ Inf 34(4):285–291. 10.3233/EFI-180221

[26] Hu XY, Young B, Santer M, Everitt H, Pearson J, Bowers H, Moore M, Little P, Pincus T, Price C, Robson T, de Barros C, Loewy J, Magee J, Geraghty AWA (2025) Self-management interventions for chronic widespread pain including fibromyalgia: a systematic review and qualitative evidence synthesis. Pain 166(3):e36–e50. 10.1097/j.pain.0000000000003379 39287095 10.1097/j.pain.0000000000003379 PMC11808693

[27] Kool MB, van Middendorp H, Lumley MA, Bijlsma JW, Geenen R (2013) Social support and invalidation by others contribute uniquely to the understanding of physical and mental health of patients with rheumatic diseases. J Health Psychol 18(1):86–95. 10.1177/1359105312436438 22363049 10.1177/1359105312436438

[28] Kusenbach M (2010) Street phenomenology: the go-along as ethnographic research tool. In: Atkinson P, Delamont S (eds) Street phenomenology: the go-along as ethnographic research tool. SAGE Publications, Inc, pp 456–484. 10.1177/146613810343007

[29] Larsson A, Feldthusen C, Mannerkorpi K (2020) Factors promoting physical activity in women with fibromyalgia: a qualitative interview study. BMJ Open 10(8):e031693. 10.1136/bmjopen-2019-031693 32784252 10.1136/bmjopen-2019-031693 PMC7418681

[30] Lazaridou A, Koulouris A, Dorado K, Chai P, Edwards R, Schreiber K (2019) The impact of a daily yoga program for women with fibromyalgia. Int J Yoga 12(3):206. 10.4103/ijoy.IJOY_72_18 31543629 10.4103/ijoy.IJOY_72_18 PMC6746047

[31] Leese C, Gupte D, Christogianni A, Higgins C, Adair P, Dall P, Cameron P, Smith BH, Colvin L (2024) Barriers and facilitators for physical activity in people living with chronic pain: a systematic review and combined analysis. Pain 165(12):2721–2732. 10.1097/j.pain.0000000000003314 38981051 10.1097/j.pain.0000000000003314

[32] Levitt HM, Bamberg M, Creswell JW, Frost DM, Josselson R, Suárez-Orozco C (2018) Journal articlereporting standards for qualitative primary, qualitative meta-analytic, and mixed methods research in psychology: the APA Publications and Communications Board task force report. Am Psychol 73:26–46. 10.1037/amp0000151 29345485 10.1037/amp0000151

[33] Lewin S, Glenton C, Munthe-Kaas H et al (2015) Using qualitative evidence in decision making for health and social interventions: an approach to assess confidence in findings from qualitative evidence syntheses (GRADE-CERQual). PLOS MED 12:e1002065. 10.1371/journal.pmed.1001895 10.1371/journal.pmed.1001895 PMC4624425 26506244

[34] Lichtenstein A, Tiosano S, Amital H (2018) The complexities of fibromyalgia and its comorbidities. Curr Opin Rheumatol 30(1):94–100. 10.1097/BOR.0000000000000464 29040155 10.1097/BOR.0000000000000464

[35] Lucini D, Giovanelli L, Bazzichi L, Bernardelli G, Pellegrino G, Filippou G, Sarzi Puttini P (2024) Tailored exercise programmes for fibromyalgia: a clinical practical guide. Clin Exp Rheumatol 42(6):1262–1271. 10.55563/clinexprheumatol/k3qldz 38910571 10.55563/clinexprheumatol/k3qldz

[36] Macfarlane GJ, Kronisch C, Dean LE, Atzeni F, Häuser W, Fluß E, Choy E, Kosek E, Amris K, Branco J, Dincer F, Leino-Arjas P, Longley K, McCarthy GM, Makri S, Perrot S, Sarzi-Puttini P, Taylor A, Jones GT (2017) EULAR revised recommendations for the management of fibromyalgia. Ann Rheum Dis 76(2):318–328. 10.1136/annrheumdis-2016-209724 27377815 10.1136/annrheumdis-2016-209724

[37] Mannerkorpi K, Gard G (2003) Physiotherapy group treatment for patients with fibromyalgia—an embodied learning process. Disabil Rehabil 25(24):1372–1380. 10.1080/09638280310001616367 14660205 10.1080/09638280310001616367

[38] Mayana KI. Association between chronic widespread pain and physical activity behaviour in people with fibromyalgia. Available at: https://salford-repository.worktribe.com/output/1335142/association-between-chronic-widespread-pain-and-physical-activity-behaviour-in-people-with-fibromyalgia

[39] Mengshoel AM, Sim J, Ahlsen B, Madden S (2018) Diagnostic experience of patients with fibromyalgia - a meta-ethnography. Chronic Illn 14(3):194–211. 10.1177/1742395317718035 28762775 10.1177/1742395317718035

[40] Moher D, Liberati A, Tetzlaff J, Altman DG, Group (2009) Preferred reporting items for systematic reviews and meta-analyses: the PRISMA statement. PLoS Med 6:e1000097. 10.1371/journal.pmed.1000097 19621072 10.1371/journal.pmed.1000097 PMC2707599

[41] Montesó-Curto P, Toussaint L, Kueny A et al (2023) Physical activity and exercise experience in Spanish and US men with fibromyalgia: a qualitative cross-cultural study. Int J Environ Res Public Health 20(18):6731. 10.3390/ijerph20186731 37754590 10.3390/ijerph20186731 PMC10531223

[42] Munthe-Kaas H, Bohren MA, Glenton C et al (2018) Applying GRADE-CERQual to qualitative evidence synthesis findings—paper 3: how to assess methodological limitations. Implement Sci 13(9). 10.1186/s13012-017-0690-9 10.1186/s13012-017-0690-9 PMC5791044 29384078

[43] Noyes J, Booth A, Lewin S et al (2018) Applying GRADE-CERQual to qualitative evidence synthesis findings—paper 6: how to assess relevance of the data. Implement Sci 13(4). 10.1186/s13012-017-0693-6 10.1186/s13012-017-0693-6 PMC5791042 29384080

[44] Núñez-Cortés R, Suso-Martí L, Almonacid-Lleida J, Salazar-Méndez J, López-Bueno R, Cruz-Montecinos C, Andersen LL, Ramírez-Vélez R, Calatayud J (2025) Optimal dose of aerobic exercise programs to reduce pain intensity and improve health status in patients with fibromyalgia: a dose-response meta-analysis. Phys Ther 105(6):pzaf057. 10.1093/ptj/pzaf057 40272395 10.1093/ptj/pzaf057

[45] Ouzzani M, Hammady H, Fedorowicz Z et al (2016) Rayyan—a web and mobile app for systematic reviews. Syst Rev 5:210. 10.1186/s13643-016-0384-4 27919275 10.1186/s13643-016-0384-4 PMC5139140

[46] Rodríguez-Almagro D, Del Moral-García M, López-Ruiz MDC, Cortés-Pérez I, Obrero-Gaitán E, Lomas-Vega R (2023) Optimal dose and type of exercise to reduce pain, anxiety and increase quality of life in patients with fibromyalgia. A systematic review with meta-analysis. Front Physiol 14:1170621. 10.3389/fphys.2023.1170621 37123268 10.3389/fphys.2023.1170621 PMC10130662

[47] Rodríguez-Domínguez ÁJ, Rebollo-Salas M, Chillón-Martínez R, Rosales-Tristancho A, Villa-Del-Pino I, Jiménez-Rejano JJ (2025) The most effective therapeutic exercises for pain intensity in women with fibromyalgia: a systematic review and network meta-analysis. Braz J Phys Ther 29(4):101226. 10.1016/j.bjpt.2025.101226 40319533 10.1016/j.bjpt.2025.101226 PMC12099910

[48] Russell D, Álvarez Gallardo IC, Wilson I et al (2018) ‘Exercise to me is a scary word’: perceptions of fatigue, sleep dysfunction, and exercise in people with fibromyalgia syndrome—a focus group study. Rheumatol Int 38(3):507–515. 10.1007/s00296-018-3932-5 29340774 10.1007/s00296-018-3932-5

[49] Sandelowski M (2015) A matter of taste: evaluating the quality of qualitative research. Nurs Inq 22:86–94. 10.1111/nin.12080 25213076 10.1111/nin.12080

[50] Sandelowski M, Barroso J (2007) Handbook for synthesizing qualitative research. Springer, New York, NY

[51] Sandelowski M, Docherty S, Emden C (1997) Qualitative metasynthesis: issues and techniques. Res Nurs Health 20:365–371. 10.1002/(sici)1098-240x(199708)20:4<365::aid-nur9>3.3.co;2-7 9256882 10.1002/(sici)1098-240x(199708)20:4<365::aid-nur9>3.0.co;2-e

[52] Sanjuan-Sánchez D, Climent-Sanz C, Patiño-Vera MDM, Gea-Sánchez M, Rubí-Carnacea F, Briones Vozmediano E (2025) “Quiero seguir en activo”: Estrategias de mujeres que padecen fibromialgia para realizar actividades cotidianas básicas, instrumentales y avanzadas. Arch Prev Riesgos Labor 28(1):31–53. 10.12961/aprl.2025.28.01.05 40029786 10.12961/aprl.2025.28.01.05

[53] Sanz-Baños Y, Pastor MÁ, Velasco L et al (2016) To walk or not to walk: insights from a qualitative description study with women suffering from fibromyalgia. Rheumatol Int 36(8):1135–1143. 10.1007/s00296-016-3459-6 26979604 10.1007/s00296-016-3459-6

[54] Sarzi-Puttini P, Giorgi V, Marotto D, Atzeni F (2020) Fibromyalgia: an update on clinical characteristics, aetiopathogenesis and treatment. Nat Rev Rheumatol 16(11):645–660. 10.1038/s41584-020-00506-w 33024295 10.1038/s41584-020-00506-w

[55] Sermenli N, Sarıtaş F, Tonga E. Perspectives on physical activity among women with fibromyalgia: a qualitative study. J Public Health. 10.1007/s10389-025-02450-z. Published online April 7, 2025

[56] Sim J, Madden S (2008) Illness experience in fibromyalgia syndrome: a metasynthesis of qualitative studies. Soc Sci Med 67(1):57–67. 10.1016/j.socscimed.2008.03.003 18423826 10.1016/j.socscimed.2008.03.003

[57] Takai (2022) Physical and mental effectiveness of a new 3-week exercise-based intervention program for fibromyalgia inpatients: interview revealed the importance of therapists’ stance. Rigakuryoho Kagaku 37(1):45–58

[58] Tong A, Flemming K, McInnes E, Oliver S, Craig J (2012) Enhancing transparency in reporting the synthesis of qualitative research: ENTREQ. BMC Med Res Methodol 12:181. 10.1186/1471-2288-12-18178 23185978 10.1186/1471-2288-12-181 PMC3552766

[59] Vancampfort D, Van Damme T, Brunner E, McGrath RL, Hemmings L, Guimaraes ME, Schuch F (2024) Dropout from exercise interventions in adults with fibromyalgia: a systematic review and meta-analysis. Arch Phys Med Rehabil 105(3):571–579. 10.1016/j.apmr.2023.06.002 37331421 10.1016/j.apmr.2023.06.002

[60] VanRavenstein, Kathryn A. Physical activity in women with fibromyalgia. Diss Med Univ S C. Published online 2014. Available at: https://www.proquest.com/openview/0024cb2809227f27c85b8f295f8eb0af/1?pq-origsite=gscholar&cbl=18750

[61] Yardley L, Morrison L, Bradbury K, Muller I (2015) The person-based approach to intervention development: application to digital health-related behavior change interventions. J Med Internet Res 17(1):e30. 10.2196/jmir.4055 25639757 10.2196/jmir.4055 PMC4327440

