# Supplementary material for: Perception and lived experience of movement in patients with fibromyalgia: a qualitative systematic review with meta-synthesis and meta-summary
Source: Clin Rheumatol. 2026 Feb 25;45(5):2437–62. doi: 10.1007/s10067-026-08005-1 (PMC13068694; doi:10.1007/s10067-026-08005-1)
Supplement: Supplementary file 1 — Supplementary Material 1 (DOCX 15.3 KB) [file 10067_2026_8005_MOESM1_ESM.docx]

**Supplementary File 1.** SPIDER approach for qualitative research (1)

| **SPIDER component** | **Definition** |
| --- | --- |
| Sample | Adults patients with diagnosis of fibromyalgia |
| Phenomenon of interest | Exercise, movement, physical activity, activity of daily living |
| Design | Interview, discussion, observation, focus groups, narration, semi structured, unstructured, structured, informal, in depth, face-to-face, personal narrative, narrative accounts, surveys and questionnaires, nursing methodology research, hermeneutics, phenomenology, storytelling, patient acuity, grounded theory, narration, thematic analysis, content analysis, questionnaire, survey |
| Evaluation | View, experience, opinion, attitude, perception, belief, feel, know, behaviour, understand, comprehension, perspective, involvement, engagement, first-person perspectives, feedback, quality of life, health-related quality-of-life, psychosocial, psychological, emotions, mental outlook, needs, living with, coping with, self-understanding, well-being, health status, patient reported outcomes, patient-centred care, healthcare, barriers and enablers, life change events, attitude, emotions, quality of life, activities of daily living, social participation, patient participation, work perceptions, occupational activity, knowledge, health knowledge, attitudes, practice, metacognition, perception, pain perception, social perception, self-concept, attitude to health, behaviour and behaviour mechanisms, experience, opinion, participation, emotional involvement, self-concept, image, feeling |
| Research type | Qualitative research, qualitative, mixed-method, multi-method, methodology, synthesis, approaches |

*Reference:*

1. Cooke A, Smith D, Booth A. Beyond PICO: the SPIDER tool for qualitative evidence syn-thesis. Qual Health Res. 2012;22. <https://doi.org/10.1177/1049732312452938>.
