# Supplementary material for: Perception and lived experience of movement in patients with fibromyalgia: a qualitative systematic review with meta-synthesis and meta-summary
Source: Clin Rheumatol. 2026 Feb 25;45(5):2437–62. doi: 10.1007/s10067-026-08005-1 (PMC13068694; doi:10.1007/s10067-026-08005-1)
Supplement: Supplementary file 2 — Supplementary Material 2 (DOCX 29.9 KB) [file 10067_2026_8005_MOESM2_ESM.docx]

**Supplementary File 2**. Search strings for each database.

| **Database** | **Search string** |
| --- | --- |
| MEDLINE  (PubMed) | (fibromyalgia OR Fibromyalgia syndrome [Mesh terms]) AND (Exercise OR Exercise [Mesh Terms] OR movement OR “physical activity” OR “activity of daily living” OR walk* OR gait OR “activity behavior*”) AND (("interview*" OR "discussion*" OR "observ*" OR "focus groups" OR "narration" OR "semistructured" OR "unstructured" OR "structured" OR "informal" OR "indepth" OR "face-to-face" OR "personal narrative" OR "personal narrative" OR "narrative accounts" OR "Surveys and Questionnaires" OR "observation" OR "Nursing Methodology Research" OR "Phenomenological" OR "Hermeneutics" OR "Patient Acuity" OR "Grounded Theory" OR "Narration" OR "Questionnaire" OR "Survey" OR "Interview" OR "Focus group") OR ("view*" OR "Experience" "experienc*" OR "attitude*" OR "perception" OR "perce*" OR "belie*" OR "feel*" OR "know*" OR "behavior*" OR "understand*" OR "comprehension" OR "perspective" OR "involvement" OR "engagement" OR "first-person perspectives" OR "feedback" OR "quality of life" OR "health-related quality-of-life" OR "psychosocial" OR "psychological" OR "emotions" OR "mental outlook" OR "needs" OR "Living" OR "living with" OR "coping with" OR "self understanding" OR "well-being" OR "health status" OR "patient reported outcomes" OR "patient-centred care" OR "healthcare" OR "barriers and enablers" OR "Life Change Events" OR "Disability" OR "Disability perc*" OR "Attitude" OR "Behavior" OR "Emotions" OR "Quality of Life" OR "Activities of Daily Living" OR "Social Participation" OR "Patient Participation" OR "Knowledge" OR "Health Knowledge, Attitudes, Practice" OR "Metacognition" OR "Perception" OR "Pain Perception" OR "Social Perception" OR "Self Concept" OR "Attitude" OR "Attitude to Health" OR "Emotions" OR "Behavior and Behavior Mechanisms" OR "Experience*" OR "Opinion*" OR "Quality of life" OR "Belie*" OR "Feel*" OR "Attitude*" OR "Participation" OR "Emotional Involvement" OR "Self-concept" OR "Self concept" OR "Image" OR "View*" OR "perspective" OR "Perception" OR "Feeling*" OR "Behavi*")) AND ("Qualitative" OR "Qualitative stud*" OR "Qualitative Research" OR "Mixed Method" OR "methodology" OR "synthesis" OR "approaches") |
| CINAHL | (fibromyalgia OR (MH "Fibromyalgia syndrome+")) AND (Exercise OR (MH Exercise+) OR movement OR "physical activity" OR "activity of daily living" OR walk* OR gait OR "activity behavior*" ) AND ((interview* OR discussion* OR observ* OR "focus groups" OR narration OR semistructured OR unstructured OR structured OR informal OR indepth OR face-to-face OR "personal narrative" OR "personal narrative" OR "narrative accounts" OR "Surveys and Questionnaires" OR observation OR "Nursing Methodology Research" OR Phenomenological OR Hermeneutics OR "Patient Acuity" OR "Grounded Theory" OR Narration OR Questionnaire OR Survey OR Interview OR "Focus group" ) OR (view* OR "Experience "experienc*"" OR attitude* OR perception OR perce* OR belie* OR feel* OR know* OR behavior* OR understand* OR comprehension OR perspective OR involvement OR engagement OR "first-person perspectives" OR feedback OR "quality of life" OR "health-related quality-of-life" OR psychosocial OR psychological OR emotions OR "mental outlook" OR needs OR Living OR "living with" OR "coping with" OR "self understanding" OR well-being OR "health status" OR "patient reported outcomes" OR "patient-centred care" OR healthcare OR "barriers and enablers" OR "Life Change Events" OR Disability OR "Disability perc*" OR Attitude OR Behavior OR Emotions OR "Quality of Life" OR "Activities of Daily Living" OR "Social Participation" OR "Patient Participation" OR Knowledge OR "Health Knowledge, Attitudes, Practice" OR Metacognition OR Perception OR "Pain Perception" OR "Social Perception" OR "Self Concept" OR Attitude OR "Attitude to Health" OR Emotions OR "Behavior and Behavior Mechanisms" OR Experience* OR Opinion* OR "Quality of life" OR Belie* OR Feel* OR Attitude* OR Participation OR "Emotional Involvement" OR Self-concept OR "Self concept" OR Image OR View* OR perspective OR Perception OR Feeling* OR Behavi* )) AND (Qualitative OR "Qualitative stud*" OR "Qualitative Research" OR "Mixed Method" OR methodology OR synthesis OR approaches ) |
| SCOPUS | (fibromyalgia OR "Fibromyalgia syndrome") AND (Exercise OR Exercise OR movement OR "physical activity" OR "activity of daily living" OR walk* OR gait OR "activity behavior*" ) AND ((interview* OR discussion* OR observ* OR "focus groups" OR narration OR semistructured OR unstructured OR structured OR informal OR indepth OR face-to-face OR "personal narrative" OR "personal narrative" OR "narrative accounts" OR "Surveys and Questionnaires" OR observation OR "Nursing Methodology Research" OR Phenomenological OR Hermeneutics OR "Patient Acuity" OR "Grounded Theory" OR Narration OR Questionnaire OR Survey OR Interview OR "Focus group" ) OR (view* OR "Experience "experienc*"" OR attitude* OR perception OR perce* OR belie* OR feel* OR know* OR behavior* OR understand* OR comprehension OR perspective OR involvement OR engagement OR "first-person perspectives" OR feedback OR "quality of life" OR "health-related quality-of-life" OR psychosocial OR psychological OR emotions OR "mental outlook" OR needs OR Living OR "living with" OR "coping with" OR "self understanding" OR well-being OR "health status" OR "patient reported outcomes" OR "patient-centred care" OR healthcare OR "barriers and enablers" OR "Life Change Events" OR Disability OR "Disability perc*" OR Attitude OR Behavior OR Emotions OR "Quality of Life" OR "Activities of Daily Living" OR "Social Participation" OR "Patient Participation" OR Knowledge OR "Health Knowledge, Attitudes, Practice" OR Metacognition OR Perception OR "Pain Perception" OR "Social Perception" OR "Self Concept" OR Attitude OR "Attitude to Health" OR Emotions OR "Behavior and Behavior Mechanisms" OR Experience* OR Opinion* OR "Quality of life" OR Belie* OR Feel* OR Attitude* OR Participation OR "Emotional Involvement" OR Self-concept OR "Self concept" OR Image OR View* OR perspective OR Perception OR Feeling* OR Behavi* )) AND (Qualitative OR "Qualitative stud*" OR "Qualitative Research" OR "Mixed Method" OR methodology OR synthesis OR approaches ) |
| Web of science | (fibromyalgia OR "Fibromyalgia syndrome") AND (Exercise OR Exercise OR movement OR "physical activity" OR "activity of daily living" OR walk* OR gait OR "activity behavior*" ) AND ((interview* OR discussion* OR observ* OR "focus groups" OR narration OR semistructured OR unstructured OR structured OR informal OR indepth OR face-to-face OR "personal narrative" OR "personal narrative" OR "narrative accounts" OR "Surveys and Questionnaires" OR observation OR "Nursing Methodology Research" OR Phenomenological OR Hermeneutics OR "Patient Acuity" OR "Grounded Theory" OR Narration OR Questionnaire OR Survey OR Interview OR "Focus group" ) OR (view* OR "Experience "experienc*"" OR attitude* OR perception OR perce* OR belie* OR feel* OR know* OR behavior* OR understand* OR comprehension OR perspective OR involvement OR engagement OR "first-person perspectives" OR feedback OR "quality of life" OR "health-related quality-of-life" OR psychosocial OR psychological OR emotions OR "mental outlook" OR needs OR Living OR "living with" OR "coping with" OR "self understanding" OR well-being OR "health status" OR "patient reported outcomes" OR "patient-centred care" OR healthcare OR "barriers and enablers" OR "Life Change Events" OR Disability OR "Disability perc*" OR Attitude OR Behavior OR Emotions OR "Quality of Life" OR "Activities of Daily Living" OR "Social Participation" OR "Patient Participation" OR Knowledge OR "Health Knowledge, Attitudes, Practice" OR Metacognition OR Perception OR "Pain Perception" OR "Social Perception" OR "Self Concept" OR Attitude OR "Attitude to Health" OR Emotions OR "Behavior and Behavior Mechanisms" OR Experience* OR Opinion* OR "Quality of life" OR Belie* OR Feel* OR Attitude* OR Participation OR "Emotional Involvement" OR Self-concept OR "Self concept" OR Image OR View* OR perspective OR Perception OR Feeling* OR Behavi* )) AND (Qualitative OR "Qualitative stud*" OR "Qualitative Research" OR "Mixed Method" OR methodology OR synthesis OR approaches ) |
| EMBASE | (fibromyalgia OR Fibromyalgia syndrome/exp) AND (Exercise OR Exercise/exp OR movement OR physical activity OR activity of daily living OR walk* OR gait OR activity behavior*) AND ((interview* OR discussion* OR observ* OR focus groups OR narration OR semistructured OR unstructured OR structured OR informal OR indepth OR face-to-face OR personal narrative OR personal narrative OR narrative accounts OR Surveys and Questionnaires OR observation OR Nursing Methodology Research OR Phenomenological OR Hermeneutics OR Patient Acuity OR Grounded Theory OR Narration OR Questionnaire OR Survey OR Interview OR Focus group) OR (view* OR Experience experienc* OR attitude* OR perception OR perce* OR belie* OR feel* OR know* OR behavior* OR understand* OR comprehension OR perspective OR involvement OR engagement OR first-person perspectives OR feedback OR quality of life OR health-related quality-of-life OR psychosocial OR psychological OR emotions OR 'mental outlook' OR needs OR Living OR living with OR coping with OR self understanding OR well-being OR health status OR patient reported outcomes OR patient-centred care OR healthcare OR barriers and enablers OR Life Change Events OR Disability OR Disability perc* OR Attitude OR Behavior OR Emotions OR Quality of Life OR Activities of Daily Living OR Social Participation OR Patient Participation OR Knowledge OR Health Knowledge, Attitudes, Practice OR Metacognition OR Perception OR Pain Perception OR Social Perception OR Self Concept OR Attitude OR Attitude to Health OR Emotions OR Behavior and Behavior Mechanisms OR Experience* OR Opinion* OR Quality of life OR Belie* OR Feel* OR Attitude* OR Participation OR Emotional Involvement OR Self-concept OR Self concept OR Image OR View* OR perspective OR Perception OR Feeling* OR Behavi*)) AND (Qualitative OR Qualitative stud* OR Qualitative Research OR Mixed Method OR methodology OR synthesis OR approaches) |
| PsycINFO  SPORTDiscus | (fibromyalgia OR exp "Fibromyalgia syndrome"/) AND (Exercise OR exp Exercise/ OR movement OR "physical activity" OR "activity of daily living" OR walk* OR gait OR "activity behavior*" ) AND ((interview* OR discussion* OR observ* OR "focus groups" OR narration OR semistructured OR unstructured OR structured OR informal OR indepth OR face-to-face OR "personal narrative" OR "personal narrative" OR "narrative accounts" OR "Surveys and Questionnaires" OR observation OR "Nursing Methodology Research" OR Phenomenological OR Hermeneutics OR "Patient Acuity" OR "Grounded Theory" OR Narration OR Questionnaire OR Survey OR Interview OR "Focus group" ) OR (view* OR "Experience "experienc*"" OR attitude* OR perception OR perce* OR belie* OR feel* OR know* OR behavior* OR understand* OR comprehension OR perspective OR involvement OR engagement OR "first-person perspectives" OR feedback OR "quality of life" OR "health-related quality-of-life" OR psychosocial OR psychological OR emotions OR "mental outlook" OR needs OR Living OR "living with" OR "coping with" OR "self understanding" OR well-being OR "health status" OR "patient reported outcomes" OR "patient-centred care" OR healthcare OR "barriers and enablers" OR "Life Change Events" OR Disability OR "Disability perc*" OR Attitude OR Behavior OR Emotions OR "Quality of Life" OR "Activities of Daily Living" OR "Social Participation" OR "Patient Participation" OR Knowledge OR "Health Knowledge, Attitudes, Practice" OR Metacognition OR Perception OR "Pain Perception" OR "Social Perception" OR "Self Concept" OR Attitude OR "Attitude to Health" OR Emotions OR "Behavior and Behavior Mechanisms" OR Experience* OR Opinion* OR "Quality of life" OR Belie* OR Feel* OR Attitude* OR Participation OR "Emotional Involvement" OR Self-concept OR "Self concept" OR Image OR View* OR perspective OR Perception OR Feeling* OR Behavi* )) AND (Qualitative OR "Qualitative stud*" OR "Qualitative Research" OR "Mixed Method" OR methodology OR synthesis OR approaches )  ("fibromyalgia" OR DE "Fibromyalgia syndrome") AND ("Exercise" OR DE "Exercise" OR "movement" OR "physical activity" OR "activity of daily living" OR "walk*" OR "gait" OR "activity behavior*") AND (("interview*" OR "discussion*" OR "observ*" OR "focus groups" OR "narration" OR "semistructured" OR "unstructured" OR "structured" OR "informal" OR "indepth" OR "face-to-face" OR "personal narrative" OR "personal narrative" OR "narrative accounts" OR "Surveys and Questionnaires" OR "observation" OR "Nursing Methodology Research" OR "Phenomenological" OR "Hermeneutics" OR "Patient Acuity" OR "Grounded Theory" OR "Narration" OR "Questionnaire" OR "Survey" OR "Interview" OR "Focus group") OR ("view*" OR "Experience experienc*""" OR "attitude*" OR "perception" OR "perce*" OR "belie*" OR "feel*" OR "know*" OR "behavior*" OR "understand*" OR "comprehension" OR "perspective" OR "involvement" OR "engagement" OR "first-person perspectives" OR "feedback" OR "quality of life" OR "health-related quality-of-life" OR "psychosocial" OR "psychological" OR "emotions" OR "mental outlook" OR "needs" OR "Living" OR "living with" OR "coping with" OR "self understanding" OR "well-being" OR "health status" OR "patient reported outcomes" OR "patient-centred care" OR "healthcare" OR "barriers and enablers" OR "Life Change Events" OR "Disability" OR "Disability perc*" OR "Attitude" OR "Behavior" OR "Emotions" OR "Quality of Life" OR "Activities of Daily Living" OR "Social Participation" OR "Patient Participation" OR "Knowledge" OR "Health Knowledge, Attitudes, Practice" OR "Metacognition" OR "Perception" OR "Pain Perception" OR "Social Perception" OR "Self Concept" OR "Attitude" OR "Attitude to Health" OR "Emotions" OR "Behavior and Behavior Mechanisms" OR "Experience*" OR "Opinion*" OR "Quality of life" OR "Belie*" OR "Feel*" OR "Attitude*" OR "Participation" OR "Emotional Involvement" OR "Self-concept" OR "Self concept" OR "Image" OR "View*" OR "perspective" OR "Perception" OR "Feeling*" OR "Behavi*")) AND ("Qualitative" OR "Qualitative stud*" OR "Qualitative Research" OR "Mixed Method" OR "methodology" OR "synthesis" OR "approaches") |

.
