# Supplementary material for: Perception and lived experience of movement in patients with fibromyalgia: a qualitative systematic review with meta-synthesis and meta-summary
Source: Clin Rheumatol. 2026 Feb 25;45(5):2437–62. doi: 10.1007/s10067-026-08005-1 (PMC13068694; doi:10.1007/s10067-026-08005-1)
Supplement: Supplementary file 3 — Supplementary Material 3 (DOCX 17.2 KB) [file 10067_2026_8005_MOESM3_ESM.docx]

**Supplementary File 3.** Certainty assessment

The GRADE-CERQual approach (1) was used to assess the certainty of the review findings. This method offers a systematic and transparent evaluation based on four components:

1. Methodological limitations, which assess concerns about the design or conduct of the primary studies contributing to a given finding (2);
2. Relevance, which examines how well the data from primary studies match the context specified in the review question (3);
3. Adequacy, which considers the richness and volume of data supporting each finding (4);
4. Coherence, which evaluates how clear and logical the link is between the primary-study data and the synthesized review finding (5).

Each component was assessed separately and rated as having “no or very minor concerns,” “minor concerns,” “moderate concerns,” or “serious concerns,” with a justification recorded for every judgement. Concerns in any component could reduce confidence in a review finding. All findings were initially assigned a rating of “high confidence,” which was then downgraded by one or two levels depending on the number and seriousness of concerns in each component. For minor or moderate concerns, the overall confidence rating was downgraded only when there were two or more such concerns within the same component (6).

GRADE-CERQual confidence assessments were carried out using the Interactive Summary of Qualitative Findings (iSoQ) online data-management tool, which supports application of this approach (5). Two reviewers (MC, GR) independently judged the confidence in each qualitative finding using this method for handling disagreements.

*References:*

1. Lewin S, Glenton C, Munthe-Kaas H, Carlsen B, Colvin CJ, Gülmezoglu M, Noyes J, Booth A, Garside R, Rashidian A. Using qualitative evidence in decision making for health and social interventions: an approach to assess confidence in findings from 704 qualitative evidence syntheses (GRADE-CERQual). PLoS Med. 2015 27;12(10):e1001895. doi: 10.1371/journal.pmed.1001895. Erratum in: PLoS Med. 2016 706 Jun 10;13(6):e1002065. doi: 10.1371/journal.pmed.1002065

2. Munthe-Kaas H, Bohren MA, Glenton C, et al. Applying GRADE-CERQual to qualitative evidence synthesis findings—paper 3: how to assess methodological limitations. Implementation Science. 2018;13(S1):9. doi:10.1186/s13012-017-0690-9

3. Noyes J, Booth A, Lewin S, et al. Applying GRADE-CERQual to qualitative evidence synthesis findings–paper 6: how to assess relevance of the data. Implementation Science. 2018;13(S1):4. doi:10.1186/s13012-017-0693-6

4. Glenton C, Carlsen B, Lewin S, et al. Applying GRADE-CERQual to qualitative evidence synthesis findings—paper 5: how to assess adequacy of data. Implementation Science. 2018;13(S1):14. doi:10.1186/s13012-017-0692-7

5. Colvin CJ, Garside R, Wainwright M, et al. Applying GRADE-CERQual to qualitative evidence synthesis findings—paper 4: how to assess coherence. Implementation Science. 2018;13(S1):13. doi:10.1186/s13012-017-0691-8

6. Wainwright M, Zahroh RI, Tunçalp Ö, et al. The use of GRADE-CERQual in qualitative evidence synthesis: an evaluation of fidelity and reporting. Health Res Policy Syst. 2023;21(1). doi:10.1186/s12961-023-00999-3
