# Supplementary material for: Perception and lived experience of movement in patients with fibromyalgia: a qualitative systematic review with meta-synthesis and meta-summary
Source: Clin Rheumatol. 2026 Feb 25;45(5):2437–62. doi: 10.1007/s10067-026-08005-1 (PMC13068694; doi:10.1007/s10067-026-08005-1)
Supplement: Supplementary file 4 — Supplementary Material 4 (DOCX 19.6 KB) [file 10067_2026_8005_MOESM4_ESM.docx]

**Supplementary File 4.** Data synthesis and analysis

Meta-summary and meta-synthesis were carried out in accordance with the methodological steps proposed by Sandelowski and Barroso (1), and were independently performed by two authors (MS, LP). The process comprised:

1. identifying the topic through repeated readings and line-by-line analysis of the included studies;
2. extracting the target results;
3. carefully editing the findings to preserve the original authors’ intent and wording;
4. clustering similar findings to determine how results from different studies related to one another;
5. abstracting findings, removing redundancies, refining statements, and explicitly retaining contradictions and ambiguities;
6. generating themes and categories from the final set of findings using a two-cycle coding approach (first inductive coding, followed by axial coding);
7. evaluating findings for similarities and differences within and across studies and synthesizing them using a constant comparative method;
8. calculating manifest inter-study frequency effect sizes (i.e., the prevalence of each finding, computed as [number of studies containing a given finding / total number of studies] × 100); and
9. calculating intra-study intensity effect sizes (i.e., the concentration of findings within each report, computed as [number of findings in a study / total number of findings] × 100).

Any disagreements arising during the process were resolved through discussion and consensus with the wider research team. Detailed themes and illustrative quotations from the meta-synthesis are presented in Supplementary File 5.

*Reference*

1. Sandelowski M, Barroso J. Handbook for Synthesizing Qualitative Research. New York:

Springer Publishing Company Inc; 2007.
