# Supplementary material for: Perception and lived experience of movement in patients with fibromyalgia: a qualitative systematic review with meta-synthesis and meta-summary
Source: Clin Rheumatol. 2026 Feb 25;45(5):2437–62. doi: 10.1007/s10067-026-08005-1 (PMC13068694; doi:10.1007/s10067-026-08005-1)
Supplement: Supplementary file 5 — Supplementary Material 5 (DOCX 87.0 KB) [file 10067_2026_8005_MOESM5_ESM.docx]

**Supplementary File 5.** Meta-synthesis process form the quotes to the themes

Theme 1: Past experiences of movement with fibromyalgia

Category 1A: Motivation and hope towards exercise

- “I come because I know it’s good for me, because if not ... those who stay in bed because of the pain ... straight to a wheelchair! If you don’t move, you get clearly worse” (Beltrán-CarrilloV.J, 2013)
- “because I have a strong need to be physically active, that’s something I feel in my body. When I get sick I still have to go out and walk a little. There’s some kind of fear in me to be lying there or to be seated…” (Larsson A, 2020)
- “I know that physical activity makes me feel better, I don’t feel good when I’m sitting at home on the couch. That won’t make me better, it’ll make me worse, and I need to be physically active a few times a week” (Larsson A, 2020)
- “Because I have a strong need to be physically active, that’s something I feel in my body. When I get sick I still have to go out and walk a little. There’s some kind of fear in me to be lying there or to be seated...” (Larsson A, 2020)
- “...I want to go there and try all the time because I long for it. I’m still climbing the walls even though it’s been 8–9 years since I became ill, it doesn’t matter. There’s a mental and physical memory…” (Larsson A, 2020)
- “...it’s important not to give up. Not feeling, ‘No I can’t’ because there’s something you can do...” (Larsson A, 2020)
- “I realized that I can do more, that I can challenge myself.” (Lazaridou A, 2019)
- “Yoga is the exercise I want to go home and do!” (Lazaridou A, 2019)
- “I guess I totally agree, but I do believe any form of exercise is good with a mental health condition” (Mayana K.I, 2021)
- “The feeling of going somewhere, leaving the house to walk or do leisure physical activity has been expressed to have a distraction effect. There is expression of feeling good following leisure physical activity” (Mayana K.I, 2021)
- "I know that If I don't do something physical to boost my cardiac. Amm I'm just gonna I'm just going to die very early” (Mayana K.I, 2021)
- “So, I mean, there have been times when I lost my jobon and going back about four years ago a business that I've worked for, they went broke, I lost my job and it really affected me

psychologically. And I know what I believe. My belief is as long as I keep working doing something. I can cope” (Mayana K.I, 2021)

- “But I'm very lucky that my family understanding am the son too I’ve got around me are extremely understanding with excellent and supportive” (Mayana K.I, 2021)
- “I want to talk with the rheumatologist to see if he prescribes me a physiotherapist to see if he can fix these legs” (Montesò-Curto P, 2023)
- “To lose weight, because there are people who have a tendency to gain weight with this disease” (Sanz‑Baños Y, 2016)
- “It is very difficult for me to walk, but tell myself to do it, and I have to go.” (Sanz‑Baños Y, 2016)
- “No, I think I would do it by myself.” (Sanz‑Baños Y, 2016)
- “What happens is that if I am tired, I am now dedicating to me. I was dedicated to everybody before. Now, I am dedicated to me... to my disease.” (Sanz‑Baños Y, 2016)
- “And there comes a time where if nobody will take away the pain, then I take it away myself somehow... I gained 15 kilos in 2 years” (Sanz‑Baños Y, 2016)
- “I go to the gym, that, you can’t imagine how I throw body into it, that’s like a weight we carry, but I push it” (Sanz‑Baños Y, 2016)
- “Of course, exercise is beneficial for health, but my priority is to lose weight...To both look good and fit into clothes.” (Sermenli N, 2025)
- “When I tried, surprisingly I could do it. I thought, ‘I can manage this,’ and followed the exercise program” (Takai N, 2022)
- “Even if I have pain, if I can enjoy today, that is enough. I am prepared to live with pain and want to live brightly,” (Takai N, 2022)
- “I could do leg raises with 4 kg weights 100 times. I don’t know how, but I did it!” (Takai N, 2022)
- “After discharge, I will pace myself, avoid overexertion, and live my own way” (Takai N, 2022)
- “I developed the habit of exercising during hospitalization and want to continue after discharge.” (Takai N, 2022)
- “I want to spend more time on my own enjoyment, not just chores.” (Takai N, 2022)
- “Patients must make efforts too.” (Takai N, 2022)
- “Yes, as long as I’m living I'm going to try my best to keep exercising.” (VanRavenstein K, 2014)
- “It (PA) is the most important part o f my FM treatment plan.” (VanRavenstein K, 2014)
- “Very important to keep up the heart and health”. (VanRavenstein K, 2014)

Category 1B: Fear and frustration about past experiences

- “...I can’t just go to any gym, or any class, it doesn’t work or I don’t. I just don’t, because I know that it doesn’t work. I have tried, been there -‘Of course you should try bodypump’, and this and that, but I mean yeah right -no, I can’t…” (Larsson A, 2020)
- “I did some swimming one time, and I pulled a muscle from my back, which gave me a trap, none of which made me have spasms all over my body that I actually dreaded going into pool again” (Mayana K.I, 2021)
- “Not any improvement, usually it’s worse. ‘I force myself but usually it causes pain. During and afterward, but usually afterward” (Montesó-Curto P, 2023)
- “I do not think it helps with my level of fatigue. I still wake up tired” (Montesó-Curto P, 2023)
- “There are moments you cannot breathe, there are times when you can walk a lot, others you cannot walk, the classic FMS pain” (Montesó-Curto P, 2023)
- “You try to change a light bulb, you raise your arm and it hurts all day” (Montesó-Curto P, 2023)
- “Exercise to me is a scary word” (Russel D, 2018)
- "Well, before you used to bend down as if you were going to stretch, like when you go to the gym. And now you can’t/.../It didn’t use to be this way...before it was all continuous" (Sanjuán-Sánchez D, 2025)
- "I can't bend down, and so I say... well, I'll kneel and wipe this down here/.../I can't do that. And I used to do that. And now I don't" (Sanjuán-Sánchez D, 2025)
- "In the shower, it's very difficult for me. I'm already very scared, I've fallen more than twice /.../ if you stumble you go straight to the floor. No reflexes...” (Sanjuán-Sánchez D, 2025)
- "I started to dry my hair and my arms fell to the sides /.../ I couldn't. I couldn't handle it. I couldn't cope with this.../.../I can't even put on a hair clip" (Sanjuán-Sánchez D, 2025)
- "And you have a lot that depends on you, and you can't/.../And everythingmbecomes so difficult/.../Even doing the dishes becomes too hard...” (Sanjuán-Sánchez D, 2025)
- "So, I'm afraid of overdoing it and getting those fainting spells I get. It's already happened to me three times or so, very recently too. And... before, I don't know if it would have happened to me, right?” (Sanjuán-Sánchez D, 2025)
- “...thinking you will not be able to do certain things, because you are unable to, with what you are; then you also create stress for yourself” (Sanz‑Baños Y, 2016)
- “It is that you can not do it... unless, of course, you take the medication again and in fact, I am already saturated with so much medication, really…” (Sanz‑Baños Y, 2016)
- “I joined Pilates class. I could only go for two days because I had a lot of joint pain. I couldn’t get up for two or three days because it was too much pain. I had incredible pain...I don’t think that I could try again.” (Sermenli N, 2025)

Category 1C: Altered body perception

- “...I’ve accepted my pain. It’s there like...and I have to try to divert it...I do things I enjoy, but I’ll have to pay for it, and that goes for both physical ac- tivity and other physical...but it’s worth it, I can take it” (Larsson A, 2020)
- “Yoga showed me that I could do more than I thought I could.” (Lazaridou A, 2019)
- “It (program) made me realize I can get back to practicing yoga while being aware of my limitations.” (Lazaridou A, 2019)
- “I surprised the heck out of myself.” (Lazaridou A, 2019)
- “You were feeling your body and trying to heal it.” (Lazaridou A, 2019)
- “I used to get angry and nag about small things, because I felt that my body did not have the strength and I couldn’t accept it.” (Mannerkorpi K, 2003)
- “I mean things have declined over the years. But I don't want it to decline where I'm sort of completely housebound. So, it's you know a bit of a fight really” (Mayana K.I, 2021)
- “The next day is generally a bit achy and especially on the knees, but it tends to improve over the week and then it gets back to the following week again. It’s just that it’s just a cycle, over the next six nights and then it’s time to cycle again” (Mayana K.I, 2021)
- “I think the fatigue effect you know, the capacity really that daytime capacity to do the amount of stuff I normally would do would and concentration" (Mayana K.I, 2021)
- "..my whole body is so tired--that I'm just exhausted all the time. I get depressed because I get so low because I feel like, “What sort of life is it?” (Mayana K.I, 2021)
- “I take the tramadol, it leaves me drowsy, and I can’t do any activity, but the next day I wake up normal and spend the day acceptable” (Montesó-Curto P, 2023)
- “My overall strength is probably down 60–70% from what it was a year ago” (Montesó-Curto P, 2023)
- “I park the car to take the kids to school and I’m already broken, I arrive at the car, and I feel my legs that I cannot take anymore” (Montesó-Curto P, 2023)
- “That you have to put into getting up and getting out to work, is horrendous...Even doing your normal everyday chores, even washing your hair and brushing your teeth is a lot of work” (Russel D, 2018)
- “And your physical self comes second. Your physical appearance” (Sanjuán-Sánchez D, 2025)
- "Many. Sudden movements, above all. Repetitive movements too. And if you're cold... you can't maintain your posture"/.../When you're trying to get back to your place, you say is that my arm? or did they use a mannequin's arm? (laughs)/.../And you stay there I'm about to go down and I realise I can't. But you can't/ /same deal when it comes to bending down” (Sanjuán-Sánchez D, 2025)
- "It's almost like you have no strength in your arms to squeeze” (Sanjuán-Sánchez D, 2025)
- "I worked, I was very active /.../ I would come home without resting. Because I haven't been lazy. I have always been a hard worker since I was 12 years old” (Sanjuán-Sánchez D, 2025)
- “It is normal that it is at work when you really notice it / / Of course, normally at work is where you notice it, because it is where you move around the most” (Sanjuán-Sánchez D, 2025)
- "Stress, I had a stress on me... very strong /.../ I felt that my mental capacities were reduced, and it was influencing me at work” (Sanjuán-Sánchez D, 2025)
- “I feel short of breath, and I do not suffer from a lung disease or anything, but I have to stop.” (Sanz‑Baños Y, 2016)
- “I thought I couldn’t move anymore, but I found I can.” (Takai N, 2022)
- “I realized my body could still move. My shoulder-blade pain lessened” (Takai N, 2022)

Theme 2: Movement during daily life with fibromyalgia: strategies, adaptations, and effects

Category 2A: Adaptation in the activity of daily living

- “I told them (at the workplace) that I can’t lift that package, I just can’t, as it’s too heavy. ‘‘You must help me’’, I say, or I just shout: ‘‘Hello, I need some help here’’. You see. I never did that before” (Mannerkorpi K, 2003)
- “Well, I have seven children but like, they’re all old now. The oldest is 32. Well, I’ve got three boys at home, but the girls are at my house everyday. So, there’s always somebody there to do something” (Mayana K.I, 2021)
- "But obviously, in respect to work and things, I had to kind of give up in that respect because it just wasn't going to happen, really" (Mayana K.I, 2021)
- " I could use a user seat when needed. So, it wasn't -actually that physically demanding, um, in comparison to other jobs. But for me, with um, fibromyalgia, it felt very demanding” (Mayana K.I, 2021)
- “After a shower, I need 15 min to recover from the effort that is involved” (Montesó-Curto P, 2023)
- “If I start peeling 4 potatoes, hair 2 and I have to wait to peel the other 2. ‘If you walk you have pain, but at 50 steps you have fatigue too” (Montesó-Curto P, 2023)
- “Have the beard on purpose, so I do not have to shave myself, because it represents a great effort for me” (Montesó-Curto P, 2023)
- "If I have to go down 10 or 12 stairs, I have to do it backwards. Because I can't. Impossible” (Sanjuán-Sánchez D, 2025)
- "I either change my activity or I go to the computer. And I'm sitting, or... Things that don't make me physically tired” (Sanjuán-Sánchez D, 2025)
- "And when I try to change position, I have to support one leg with the other and leverage myself to change position. I have to lever myself up and then I say...one, two and three. And I change” (Sanjuán-Sánchez D, 2025)
- “And my nails... someone else must clip them"/.../ "I can't clip my toenails” (Sanjuán-Sánchez D, 2025)
- "I have to have it done" [she needs her nails clipped] (Sanjuán-Sánchez D, 2025)
- "And before I used to reach my back -soaping myself in the shower with a sponge- And now I can't reach it /.../ Now I take a towel on both sides, and I get by “(Sanjuán-Sánchez D, 2025)
- "I have a stick thing for my back, but yes, yes... it's hard for me/.../You do change things, a lot" - to scratch/ shower/.../I have put a handle in the bath and in the shower/.../If I didn't have a shower, I would have had to take the bathtub out/.../As I have a bath and shower at home, when I feel a bit agile I use the bath” (Sanjuán-Sánchez D, 2025)
- “At home I have a shower tray” (Sanjuán-Sánchez D, 2025)
- "In the bathroom I have a bathtub, and I have to change it to a shower tray because it is very difficult for me to get into the bathtub /.../ The only thing I have bought is for...under the bathtub so that I don't slip” (Sanjuán-Sánchez D, 2025)
- “But I don't bathe, because I can't. I can't sit in the bathtub. Because when I've tried to take a relaxing bath, then I couldn't get up/.../But for sure, for sure, for sure. Because in the bathtub, I need to be sure to lift my foot, my leg to get in and out. When I feel insecure, when I see that I don't feel safe, that I might fall, I use the shower” (Sanjuán-Sánchez D, 2025)
- “And now I go to the hairdresser more often. Maybe just to wash my head and that's all” (Sanjuán-Sánchez D, 2025)
- "You don't dress the same, It's very important/.../It's not the same to wear a cute little heel as it is to wear a little thing like that.../.../The shoe, I've always worn heels like that. And now I have to wear them like this or nothing. Because I can't take a step/.../I just couldn't walk when I put them on" - the high heels “(Sanjuán-Sánchez D, 2025)
- "It limits you a lot, in terms of putting on different shoes” (Sanjuán-Sánchez D, 2025)
- "I haven't had to change anything about my clothes and so on /.../ Shoes and so on, yes. I don't know if it's the same, I've had surgery on one foot, I had Morton's neuroma” (Sanjuán-Sánchez D, 2025)
- "I had to wear high heels, be dressed up and all that /.../ I have changed to more comfortable, less flirtatious, more hippie” (Sanjuán-Sánchez D, 2025)
- "My strategy is my trolley [Uses the trolley to load better] /.../ Apart from this bag, and the other handbag. So, I've brought my trolley. Because no, otherwise I wouldn't be able to. My trolley is my companion” (Sanjuán-Sánchez D, 2025)
- "Now I've bought one of those potato peelers that you squeeze out in circles like that/.../You go and find your own comfort/.../The Thermomix, the potato peeler, the floor scrubber” (Sanjuán-Sánchez D, 2025)
- “Well, now I've bought a potato cutter/.../I've also bought that potato cutter to make slices. Ah... the lettuce squeegee/.../ The steamer is the only thing I've bought because that's what it's for, to clean the tiles and all that” (Sanjuán-Sánchez D, 2025)
- "But I also do...I bought a mop with steam. Because well, I had no strength in my legs and I couldn't walk/.../I couldn't scrub... I
  couldn't scrub. So I buy things to see how I feel” (Sanjuán-Sánchez D, 2025)
- "Cans... Well, I open them. But I have a trick, I tap the lid all around and it's already open” (Sanjuán-Sánchez D, 2025)
- “To open the jars I use a green scouring pad. And then that hooks and lets me open it better/.../To open cans, I have my own
  method- if her son and husband are not there- with the corkscrew that I bought” (Sanjuán-Sánchez D, 2025)
- “I used to peel potatoes with a normal knife. And now I've had to buy one of those good ones” (Sanjuán-Sánchez D, 2025)
- "Washer-dryer and dishwasher would be useful..../.../I have to wash with hot water. Cold water then hurts my fingers and everything/.../The dishwasher, especially, would be good for me. The dishes, the pots and pans, all that... it's hard for me. It's very hard for me” (Sanjuán-Sánchez D, 2025)
- “Before, maybe I'd finish eating and I'd do the dishes. Well, I use the dishwasher more” (Sanjuán-Sánchez D, 2025)
- “I have a mop with a pedal” (Sanjuán-Sánchez D, 2025)
- “I can't bend down and I say...Well, I'll kneel down and clean this down here” (Sanjuán-Sánchez D, 2025)
- "I already think about comfort. For example, carpets. I don't use carpets anymore because then I don't have to clean them” (Sanjuán-Sánchez D, 2025)
- "My husband has to do everything. And I can't handle the heavy things, I can't. Thank God my husband does it all for me. Thank God my husband does everything for me. Because I can't/.../When it comes to weight, a lot of weight is impossible” (Sanjuán-Sánchez D, 2025)
- "Having to live with this mess makes me a bit upset, but my husband and my son are very understanding and don't say anything to me, they don't care if it's clean or not, but they won't clean it themselves, but they won't tell me to clean it either” (Sanjuán-Sánchez D, 2025)
- “And day-to-day life? Well, you run your house as best you can” (Sanjuán-Sánchez D, 2025)
- "Because I don't clean the kitchen and so on. But I don't do it. First I try” (Sanjuán-Sánchez D, 2025)
- "When I work, yes [there are movements that bother her], because, even though my lower back hurts, I bend down well I bend
  my knees well and do what I have to do. You have to bend down with your knees bent. And if you bend down like that it's bad” (Sanjuán-Sánchez D, 2025)
- "Well, as you can well look, I try to sit well, but if my leg hurts, I stretch it, I move it, since no one can see me behind the counter (laughs). If my right arm hurts a lot, I give the papers with my left arm / / I go around, depending on the side that hurts more or less, I put myself on one side or the other” (Sanjuán-Sánchez D, 2025)
- “Only when my knees attack a lot, then I use the knee pads” (Sanjuán-Sánchez D, 2025)
- "And there are times when I can't get up from my chair. I go there, I don't get up... so I turn back - in a work context - I start working at seven in the morning until three in the afternoon, and I can't stop. Because the moment I stop I can't go on” (Sanjuán-Sánchez D, 2025)
- "So, if I am fortunate enough to be married and have a child. And my husband works, and my son works, then it is not necessary for me to work” (Sanjuán-Sánchez D, 2025)
- “Basically, on this, my backing is my 22-year old daughter.” (Sanz‑Baños Y, 2016)
- “For me... wearing a pair of good shoes for walking” (Sanz‑Baños Y, 2016)
- “When my shoulders and upper arms hurt, I rotated them 50 times morning and evening, and the pain went away.” (Takai N, 2022)

Category 2B: Fatigue management

- “IP: I don’t care about vacuum cleaning every week, even if I might feel that this is one of the days when I could manage it for a while. I don’t care about it if I don’t feel like doing it, as I can always do it the next day. // . . ..// . I no longer stress about small things. Not at all. I say no to things that I do not want to do, baby-sitting or going shopping. I don’t force myself like I used to. At that time, it was as if I had to, I had to, I had to. Well, I don’t have to!” (Mannerkorpi K, 2003)
- “IP: I have learnt to accept that I have this illness, fibromyalgia. I will never get completely well, but I can get better or worse. It goes in waves, you see, but I have accepted it. And to take each day as it comes, to try to figure out in the morning when
  I get up how the day is going to be. If I can manage a given task, that’s good. If I can’t, I leave it until the next day “ (Mannerkorpi K, 2003)
- “And when I got tired, I did actually stop - amm I usually feel very achy and. I will generally have a shower and get into my gym jams and just sit and take my painkillers” (Mayana K.I, 2021)
- “Um, gets aggravated, it’s-it’s the extreme. So, if I don’t do any a recise or any-any movement, it -um, I get very stiff and achy. But if I do too much exercise. I can leave myself bed-bound for days” (Mayana K.I, 2021)
- “Last time I was cycling. I knew I was tired, and I knew if I kept on, I put myself into Probably a flare-up if I wasn't Careful- And if I've done slightly too much or I'm not feeling well, then the weekend is basically umm housebound. I basically just rest” (Mayana K.I, 2021)
- “Even though like we are only limited to like a 20-minute break every like within a seven-hour shift, I say I am allowed to if I am feeling bad to stop and just take a sit down. So, if a day is ever really bad, then there is someone willing to swap some stuff around to cover my shift” (Mayana K.I, 2021)
- "I know I'm very lucky. The people I work for, they are very accommodating. if I'm not feeling too good It's a case of stop working if I'm tired, it's gone and rest.” (Mayana K.I, 2021)
- “I ride a bicycle for 20 min; I take a couple of laps before and after” (Montesó-Curto P, 2023)
- “I run on a treadmill for 20–25 min. It is a low-key exercise, so it does not intensify or contribute to more pain” (Montesó-Curto P, 2023)
- “In fact, I was recommended that before I get tired, I should calculate when I get fatigued, and before that I should stop. The problem is that I must stop every 15 min, but I try to force myself a little to improve and try to get to 20 min.” (Montesó-Curto P, 2023)
- “On a good day, you must watch yourself that you do not too much with that good day. You must cut back on what you do otherwise… you pay for it 2 o 3 days down the road. I realized that I had two options: to do nothing and stay at home complaining or, even if I felt tired and with pain, but living my life. A while ago, in the hospital we made a study about our heart rate and how long we could walk, for me it was very strong rhythm, I tried to do 2 or 4 days. If I do 20 or 30 min, then I have to be resting triple.” (Montesó-Curto P, 2023)
- “You really have to taper it, moderation” (Montesó-Curto P, 2023)
- “In theory, you do not have to get tired, so you do not have to rest” (Montesó-Curto P, 2023)
- “you have to make choices all the time and I think that’s the big difference between not having fibromyalgia and having fibromyalgia. If you were the normal ‘me’ you wouldn’t have to make the choice. She [referring to her ‘other’/‘normal’ self] would clean the house, go to the exercise class, go out for dinner,... but the ‘me’ now? My mind has to go ‘do I hoover and do the dishes, or do I go out for dinner?’ What do I do?” (Russel D, 2018)
- “Look, today I'll do one thing and tomorrow I'll do something else” (Sanjuán-Sánchez D, 2025)
- “Today I'm going to clean as before, (forgets), but of course after a while you're all like puff...and you have to stop” (Sanjuán-Sánchez D, 2025)
- "Well, instead of doing 40 hours I'm going to ask if I can do 30, because at night when I arrive, I'm tired” (Sanjuán-Sánchez D, 2025)
- “...Because you overcome, if you learn, you are taking a different way, but you have to be a little selfish... First you and then you and then you” (Sanz‑Baños Y, 2016)
- “I’ve taken my life like this: first Me and Me; because if not... learning to say “no” first, for me…” (Sanz‑Baños Y, 2016)
- “When I rest one day, the next day I can do a bit more. I understood the importance of resting.” (Takai N, 2022)
- “Now I can accept not doing everything 100%. Even 50% or 30% is fine. I can allow myself to rest.” (Takai N, 2022)
- “Exercise improves my pain, unless I do too much, and then it makes it worse.” (Takai N, 2022)
- “I tried yoga, but my symptoms were terrible, but I find I can walk and I feel great.” (VanRavenstein K, 2014)

Category 2C: Integration of movement during the day

- “Even now I do half hour of yoga in the morning, I notice a big difference in that day, I feel more alert.” (Lazaridou A, 2019)
- “The breathing exercises come in handy when stuck in traffic.” (Lazaridou A, 2019)
- “I need to do it at least every other day.” (Lazaridou A, 2019)
- “I do not have an exercise program, but at home I have stairs. . . I am up and down those about 15 or 17 times a day.” (Montesó-Curto P, 2023)
- “Stretching is painful but it feels better after and then it comes back. I do that 5–10 times a day” (Montesó-Curto P, 2023)
- “I go up and down steps, 15 to 17 times a day, then do my own stretching. I also do some yard work, shopping and driving the car. That’s all, I have no program” (Montesó-Curto P, 2023)
- “I miss it [exercise] badly and I keep trying (voice breaking and becoming emotional)” (Russel D, 2018)
- “The foothold it is very important” (Sanz‑Baños Y, 2016)
- “Sometimes I can get some exercise before dinner or at lunch break and go out like that.” (Sermenli N, 2025)
- “After having tea at the summer house, we would go for an evening walk every evening...” (Sermenli N, 2025)
- “Actually, after taking my child to school, I can return home a few blocks away” (Sermenli N, 2025)
- “I can think of a short period of time like ten minutes as walking and add it to my routine... Maybe I’m setting the time limit too high and I’m overthinking it and giving up.” (Sermenli N, 2025)
- “I’m going to keep doing what I’m doing for now, then slowly increase time and intensity.” (VanRavenstein K, 2014)
- “I still exercise, but I do it indoors. It’s not as much fun, but it’s worth it.” (VanRavenstein K, 2014)

Category 2D: Positive effects

- “... physical exercise helps a lot because before I had a lot of pain; if I do a little, there’s relief. Physical exercise is necessary to improve daily life.” (Cavaliere A, 2010)
- “... besides the physical benefits, I think it improves our self-esteem.” (Cavaliere A, 2010)
- “... I learned to breathe—something we didn’t know—learned at home to bend to pick something up under the sink; I mean, it’s related, because my health improved a lot, things I used to do wrong besides the fibro.” (Cavaliera A, 2010)
- “I feel more energy,” (Beltrán-Carillo V.J, 2013)
- “Now I have more mobility,” (Beltrán-Carrillo V.J, 2013)
- “I feel more agile” (Beltrán-Carrillo V.J, 2013)
- “I became more conscious of my breathing and my body.” (Lazaridou A, 2019)
- “It (yoga) helps to get the body moving again.” (Lazaridou A, 2019)
- “...I am able to keep going during the day, I have more stamina.” (Lazaridou A, 2019)
- “The breathing increased my lung capacity.” (Lazaridou A, 2019)
- “I sleep deeper, my sleep quality is better!” (Lazaridou A, 2019)
- “Oh, I am lighter.” (Lazaridou A, 2019)
- “There was no elephant sitting on my shoulder.” (Lazaridou A, 2019)
- “Warrior pose – It felt like it was stretching everything.” (Lazaridou A, 2019)
- “A step back from stress and pain.” (Lazaridou A, 2019)
- “I had a feeling of letting go and relaxing.” (Lazaridou A, 2019)
- “I think I felt happier.” (Lazaridou A, 2019)
- “I felt better about myself because I was doing something to improve my pain symptoms.” (Lazaridou A, 2019)
- “I gained confidence in myself and my abilities.” (Lazaridou A, 2019)
- “It (yoga program) gave me some goals.” (Lazaridou A, 2019)
- “With the study I felt stronger.” (Lazaridou A, 2019)
- “The pool training has been relaxing and very pleasant. And it takes away . . . you see, when you have this fibromyalgia pain and you relax, the pain diminishes a bit, I think, every time” (Mannerkorpi K, 2003)
- “ Just the warmth of the water helped you to move into a lower gear. And it was not stressful, everything was done in slow motion, so you moved into a lower gear, it was very noticeable. And the exercises . . ., there were no stressful exercises, they were pleasant all the time. Just that made the hour . . ., yes, it was very peaceful. There was no rush.” (Mannerkorpi K, 2003)
- “And you felt that you couldn’t manage it. Here I could do the things and I felt good about being able to keep up and having the strength to do these movements and sometimes to put a bit more into it. I also found this very positive.” (Mannerkorpi K, 2003)
- “I feel that I have more strength in my body. I am not healthy but I feel much better with this low-intensity exercise, stronger. This regular low-intensity training is better than nothing, not too high or too low. It is the level that is suitable for me as I am now. I feel strong.” (Mannerkorpi K, 2003)
- “The pool training has led to my learning not to rush. I am calmer, to put it simply; in the past, I stressed and rushed. Yes, it’s a question of stress, stress initiates pain. So it has been enormously useful, enormously, I cannot emphasize how important it has been. Very important.” (Mannerkorpi K, 2003)
- “Yes, I have, for example, started taking life more calmly, that’s something I have learnt. To stop stressing, to learn to take it calmly, learn to handle it, I don’t know how to explain it.” (Mannerkorpi K, 2003)
- “It has done so much for my self-esteem. I feel like a new person.” (Mannerkorpi K, 2003)
- “ No, the pain is not different, it isn’t. It can be just as painful, but I can take pain better nowadays. I can accept it in a different way. I can try to ignore it a little, to shove it out a little.” (Mannerkorpi K, 2003)
- “Um, immediately after, pretty positive 'cause you get the sort of like endorphin but- also really tired” (Mayana K.I, 2021)
- “I did find it walking beneficial, not hugely, but yes, beneficial. I don't think it's improved the pain it improved my stamina” (Mayana K.I, 2021)
- “I’ve had CBT, yes. Uh, it’s helped to a point…I did do mindfulness, I did a bit of breathing techniques, um, which helped to a point. It certainly helped with the anxiety.”(Mayana K.I, 2021)
- “I have a 4-year-old dog and I leave the house 1 or 2 times a day when I am well. There are days when I can’t, but I admit that it helps me to get out of the house and to talk to some people I meet” (Montesó-Curto P, 2023)
- “I’m tired, but I feel better. Physically I’m worse, but mentally I’m better” “(Montesó-Curto P, 2023)
- “One must force oneself to do something, so the mind becomes distracted” “(Montesó-Curto P, 2023)
- “If you’re busy, you’re less focused on pain and you feel better” (Montesó-Curto P, 2023)
- “I feel more comfortable with myself.” (Sanz‑Baños Y, 2016)
- “I feel that I can face the disease.” (Sanz‑Baños Y, 2016)
- “Walking is great because it seems that you are rusty and when you start you feel like the battery is being super-charged” (Sanz‑Baños Y, 2016)
- “Because it helps us to not be stiff, to not stand there... and you will become increasingly stiff, in a heap” (Sanz‑Baños Y, 2016)
- “As you start walking you start to heat up the muscles and it is like removing the pain” (Sanz‑Baños Y, 2016)
- “The first [advantage] is that I go out, I have that time for myself” (Sanz‑Baños Y, 2016)
- “To me, yoga brings me peace and, above all, stretching is great, stretching is great for me” (Sanz‑Baños Y, 2016)
- “When I exercised, I woke up less tired, had more energy during the day and my mood was better.” (Sermenli N, 2025)
- “The best thing was learning how to relax my stiff body. Until now, I was always tensing.” (Takai N, 2022)
- “The pain hasn’t changed much, maybe slightly, but now I know how to control it.” (Takai N, 2022)
- “I regained stamina. I was surprised: ‘I can move this much!’” (Takai N, 2022)
- “I discovered that even with pain, moving makes me feel better.” (Takai N, 2022)
- “Now I can find hope for life” (Takai N, 2022)
- “Pain became milder.” (Takai N, 2022)
- “The regular rhythm of inpatient life was good.” (Takai N, 2022)
- “I managed to get through it.” (Takai N, 2022)
- “Although I still have pain, I regained some movement and can do more than before.” (Takai N, 2022)
- “Feels like something is pulling when I start, but once I calm down, it is better.” (VanRavenstein K, 2014)

Category 2E: Negative effects

- “I guess it (activity-induced syntoms) is like a hangover, I don’t really know how that is, but… You sort of drink and drink, and everything feel so good and the day after you’re completely knocked out. Yes, you can compare it to that…” (Larsson A, 2020)
- “I tried doing yoga a few months back. I did it for a day and then I couldn't kneel for about a week, I was in so much pain” (Mayana K.I, 2021)
- “In all honesty wrecked!! everything hurts. I just ache. So, I come home and have a hot shower, get rest, take my painkillers. And quite often just go to bed” (Mayana K.I, 2021)
- “I went to a salsa class on Wednesday umm, and I made through most of the class, I was a bit stumbling in places. But the next day I was extremely tired. And then I sort of crushed up the rest of the afternoon, but I still suffer for the next day” (Mayana K.I, 2021)
- “I try to take 20 min into 45, then there is fatigue and brutal joint pain for the rest of the day and into the next day. . . More labor that I do the worse my cramps are. It hurts and when it hurts you stop” (Montesó-Curto P, 2023)
- “This weekend I was running a chainsaw and sweating a ton and just trying to work normal. . .and I was working with a lawn mower for 1 h and then I was sick for two weeks” (Montesó-Curto P, 2023)
- “It actually makes the fatigue and pain worst” (Montesó-Curto P, 2023)
- “I like to do walking and because I need to lose weight, but the more I do the more I hurt” (Montesó-Curto P, 2023)
- “The more I do the more I hurt. Then you do not walk, and you do not do things because you are hurt. It is a big snowball effect. I could not do anything they wanted me to do (yoga). It was very hard, especially for the joints” (Montesó-Curto P, 2023)
- “If I walk 100 m to take a coffee with a friend when I return home, I lie down in bed all day” (Montesó-Curto P, 2023)
- “After the minimum exercise I feel worse” (Montesó-Curto P, 2023)
- “You get a day when you’re not just too bad you would be inclined to do a bit of weeding the garden or something or clean cupboards, then a day or two later you really suffer for it.” (Russel D, 2018)
- “Well, for the next 3 days I’m not going to be able to move from the bed” (Russel D, 2018)
- “Then a day or two later you really suffer for it” (Russel D, 2018)
- "Walking, going for a walk, then my back suffers greatly from it” (Sanjuán-Sánchez D, 2025)
- “[after cooking] I had such a pain in my hips that I couldn't even walk” (Sanjuán-Sánchez D, 2025)
- "The iron... fatal. Ouch, very bad. Very bad. When I iron, I don't let it pile up because my arms and neck hurt a lot. Because you have to use a bit of strength to iron” (Sanjuán-Sánchez D, 2025)
- "The daily life of cleaning the house, I also notice I get exhausted” (Sanjuán-Sánchez D, 2025)
- "If you force yourself a little bit, for example, cleaning, then 2-3 days later you are in bed” (Sanjuán-Sánchez D, 2025)
- "It is clear to me that I will pay for the day I go too far” (Sanjuán-Sánchez D, 2025)
- “Besides, I worked in a kindergarten kitchen, I made food for forty children, I took pots, I took this I used to say: I'm exhausted, I'm exhausted (snorts)” (Sanjuán-Sánchez D, 2025)
- "At work, I felt exhausted at work, that's why I also realised it because I liked my job, but there came a time when physically” (Sanjuán-Sánchez D, 2025)
- "I was very rough at work (laughs). It demanded a lot from me, the work was at a physical level and (blows) there came a time one day when I had an anxiety crisis and well, I exploded” (Sanjuán-Sánchez D, 2025)
- “Walking does not help fibromyalgia, that is, it is not beneficial as, I do not know... Maybe, for instance, to me I see more benefits lying in bed than walking.” (Sanz‑Baños Y, 2016)
- “For me walking has no advantages” (Sanz‑Baños Y, 2016)
- “And then, above all, exhaustion, tiredness, fatigue. When I get home, I do not feel like doing anything, I have to go to rest…” (Sanz‑Baños Y, 2016)
- “As happens to my colleagues... knee pain, foot pain, low back pain and a lot of hip pain…” (Sanz‑Baños Y, 2016)
- “I have been without medication many times in my life: pregnancy, delivery, postpartum, etc. Even sometimes voluntar-
  ily because sometimes the medication has more side-effects than benefits” (Sanz‑Baños Y, 2016)
- “I bought a bike one year ago and only used it twice because I was sore and out o f breath.” (VanRavenstein K, 2014)

Theme 3: Barriers to movement: personal, environmental, and relational

Category 3A: Personal barriers

- “I was okay for the first part, but when I had to get on my knee I had trouble.” (Lazaridou A, 2019)
- “SometimesI say it takes a sometimes the pain is that bad. Just trying to put any kind of movement or weight. That is just been impossible.” (Mayana K.I, 2021)
- "It's not necessarily pain. Um, I get like really bad fatigue with, umm. So, if I do too much, uh, too many chores-- As I was cleaning my room ready for university the other day -and I did about an hour and a half of like solid tidying and hoovering, and then I had to take a nap for four hours” (Mayana K.I, 2021)
- “Usually like you sometimes feel like just very fatigued. It’s like just I don’t know don't have the energy to pretty much get up sometime even if I'm being idle, I'll just midway through the day just become very fatigued. I was to go into town, do some chores, but you know, I just haven’t had the energy to do that.” (Mayana K.I, 2021)
- “I think the fatigue effect you know, the capacity really that daytime capacity to do the amount of stuff I normally would do and concentration. It’s difficult at times. But fatigue certainly makes that difficult” (Mayana K.I, 2021)
- “There are times where I’m so exhausted in the morning. And if I go back to sleep, it’s difficult when to wake up. So, but I don’t I don’t sleep deeply either when I’m asleep” (Mayana K.I, 2021)
- “And I just feel guilty all the time because they’re my kids. I should be albe to do stuff for them. But I’m just constantly - - All I wanna do is sleep. I try and do anything; I just feel like I’m falling asleep” (Mayana K.I, 2021)
- “My husband tends to do most stufbecause he gets sick of hearing me, like-puffing and puffing in pain and crying when I- - I try and do, like, just put in loads of washing machine- in the washing machine and stuff. But I get so, like, tired from just going up and down the stairs and then bending-over to get to the washing machine. It just hurts and then obviously with the jumping and stuff like that, I can’t really be cooking and that” (Mayana K.I, 2021)
- "I think mainly because it focused on, uh, the sort of, um, like mental health, psychological si de of things. That's lessened- -the amount of stress that I feel because I'm able to get -myself out of recognise, uh, negative thinking patterns and get myself-out of the sort of holes that I used to fall into. Because with, um, fibromyalgia, obviously, being in pain all the time can make you really, really depressed and -so-. I was in pain, so I didn't do anything. Uh- - because I wasn't doing anything-I was in pain. It just sort of perpetuates that cycle, and I just already used to this. Whereas now, I have a lot of, uh -do-- you-you have this thing called a-a cap map, where you essentially map out every single behavioural pattern and sort of those thoughts focus you get into and then you can visually point out exits. So, I have that stuck on my wall, and it helps me get out of, um, that" (Mayana K.I, 2021)
- “No, no medications anymore. I came off the medications. As soon as I went on them, really because I found that it was really affecting everything. Like I was slower, I was slurred. They just felt like they were doing more damage than good. And in fact, they felt like they were wearing off” (Mayana K.I, 2021)
- “I take amitriptyline and I generally have to go and lie down in the dark and have to sleep for it to pass. If I trying to work really it generally just lingers around” (Mayana K.I, 2021)
- “Sometimes it does, sometimes it doesn’t depend on the level of pain” (Mayana K.I, 2021)
- “I can’t take it during the day though. I can only take it at night. Yeah. Because it just tends to make me a little bit foggy” (Mayana K.I, 2021)
- " So, the plan was to become a detective. I had a private detective. I had done all the course and everything. However, I could not go forward with that because driving was terrible. I was very dizzy, I felt very nauseous. I felt terrible even for driving like a small distance so I could not fulfil that role” (Mayana K.I, 2021)
- “ liked cycling a lot, but now with vertigo, I had to leave it” (Montesó-Curto P, 2023)
- “I think swimming is the best but now I can’t do any exercise at all” (Montesó-Curto P, 2023)
- “I tried to do everything, but after 10 min walking, I cannot take it anymore” (Montesó-Curto P, 2023)
- “And I say yes, I am hooked on opiates. I tried to stop the medication, I told him everything and I gave up everything and I couldn’t, in 3 days I needed it and I had a withdrawal that I couldn’t with. And then, after all, you’re useless, you’re lying in bed because you’re too high to go out, to be able to talk to people” (Montesó-Curto P, 2023)
- “I’ve gone to pick up the girls at a loss and could barely talk. I’ve been through 3 pain clinics, and I can assure you that there is nothing that takes away the pain. Morphine patches 100 mg. for 2 years and they don’t take away the pain. I eliminated them myself for 8 months by cutting off a tiny bit every day because otherwise I had to go to the hospital to take methadone and it didn’t make any sense” (Montesó-Curto P, 2023)
- “I had to give up everything I was doing during all these years. I was a jumper coach, I had been in the high-performance center, I had been runner-up in Spain 2 times, third in Spain in jumps with pole-vaulting, then after 7 years without jumping, I was 3rd in Catalonia. With the medication I take, I can’t do any physical activity. At first, I was taking Palexia (tapentadol), but I was very nauseous, and they changed it to tramadol. They gave me tramadol intravenous in the hospital because the oral medication did nothing for me” (Montesó-Curto P, 2023)
- “The more physical labor that I do the worse my cramps are” (Montesó-Curto P, 2023)
- “Maybe swimming is what worked best to channel anxiety, but now I cannot channel it with anything” (Montesó-Curto P, 2023)
- “I struggle with exercise now. I struggle to walk to the end of my street. I struggle to walk over the road... so my exercise consists of getting out of bed [and] getting my body down the stairs.” (Russel D, 2018)
- "Pain doesn't let me live! I can't carry on with my life. It limits me greatly” (Sanjuán-Sánchez D, 2025)
- "What limits you is... constant pain” (Sanjuán-Sánchez D, 2025)
- "The terrible pains... It's that... look, not even the stairs/.../I couldn't even walk because of the pain, it was horrible” (Sanjuán-Sánchez D, 2025)
- "I can't move my hands; I can't handle them. Any friction causes me pain” (Sanjuán-Sánchez D, 2025)
- "Going down the stairs. It's very hard for me. I can't do it. And if one day the lift breaks down, I'm left in the street waiting for the technician to come/.../Bending down and going up and down a staircase to do something... fatal too/.../The journey is unbearable for me. Because of the pain I have, I end up dying of pain” (Sanjuán-Sánchez D, 2025)
- "The same as when you have your make-up done. [Refers to having her arm up and down]” (Sanjuán-Sánchez D, 2025)
- "There are people who can't wash their hair/.../and people who can't take a shower. They need help” (Sanjuán-Sánchez D, 2025)
- "Using the toilet. That becomes very hard” (Sanjuán-Sánchez D, 2025)
- "When it comes to putting on my shoes. I notice that it's harder for me to put on shoes now than before” (Sanjuán-Sánchez D, 2025)
- "And always instead of going out in the morning to go shopping or anything else, to do a few errands, I just can't stand my soul” (Sanjuán-Sánchez D, 2025)
- “I couldn't even sweep with the knife” (Sanjuán-Sánchez D, 2025)
- "A basin, I can't even put it on top of the kitchen table” (Sanjuán-Sánchez D, 2025)
- "For example, this gesture, if I do it like this, as long as it's tolerable, I hold my shoulder, but of course when I'm finished, if I try to force myself more than I should, for example cleaning two panes of glass, so that we understand each other, then I pay for it in spades” (Sanjuán-Sánchez D, 2025)
- "I mean, me wiping with the cloth and wringing it out, that gesture itself, well, it's really painful and (thinks) and my back, I can't bend down well, of course, it affects me... it limits me, it limits me a lot if...” (Sanjuán-Sánchez D, 2025)
- "I drop the broom and I can't open a bottle of water” (Sanjuán-Sánchez D, 2025)
- “So, I try to bend down, take out the pots and…I can’t. I can’t” (Sanjuán-Sánchez D, 2025)
- "Cleaning windows...or the upper parts. The ceiling... Cleaning the kitchen and all this.... I can't/.../ Upper areas, glass and things like that, it's impossible” (Sanjuán-Sánchez D, 2025)
- "If I try to spend time cleaning or anything like that... Bending down, trying to look for something or getting dow on my knees, that's unthinkable now” (Sanjuán-Sánchez D, 2025)
- "When housekeeping I feel very limited because I am a very active person and I like to... I am obsessive about cleaning, and I used to spend part of my life cleaning and now I don't, now I can't” (Sanjuán-Sánchez D, 2025)
- "The first thing the doctor told me is to clean under the furniture while sitting down, but not even sitting on the stool” (Sanjuán-Sánchez D, 2025)
- “You look for a chair, you put the basin on the chair and then you go... stumbling around.” (Sanjuán-Sánchez D, 2025)
- "Now first you bend down, bend your knees and then when you get up and say: mare meua(my god), now how do I get up” (Sanjuán-Sánchez D, 2025)
- “I have cleaned the oven in my kitchen while crying in pain, sitting on the stool” (Sanjuán-Sánchez D, 2025)
- "I was late for work, a lot of stress...” (Sanjuán-Sánchez D, 2025)
- "Stress and work affected me a lot” (Sanjuán-Sánchez D, 2025)
- "Horrible pains /.../ I couldn't bend down” (Sanjuán-Sánchez D, 2025)
- "When I was working at the fairs is when I started to get the most pain. My shoulders, my arms...” (Sanjuán-Sánchez D, 2025)
- "I also lost my job. The pains were deadly. I couldn't stand it. It was…it was everything. And I can stand the pain. But it was horrible /.../ At work, the pains were deadly” (Sanjuán-Sánchez D, 2025)
- "I have been on sick leave for a year. Yes, because of fibromyalgia and everything that goes with it, psychologically and...” (Sanjuán-Sánchez D, 2025)
- "No [work], I have an absolute disability” (Sanjuán-Sánchez D, 2025)
- "I have been retired since 2003 due to absolute disability” (Sanjuán-Sánchez D, 2025)
- "No, I don't work, I have a disability, I have a pension” (Sanjuán-Sánchez D, 2025)
- "I can't work. I can't. I can't.” (Sanjuán-Sánchez D, 2025)
- "Imagine being in a life where it wasn't like that [being able to afford not to work]. If I had no balls left but to work. Well then... I wouldn't be able to work” (Sanjuán-Sánchez D, 2025)
- "In the future I won't be able to work, and life... I think about lying down all day long and well.... /.../ The first thing that worries me is that I can't work. And the future, of course I worry about what is going to happen in the future” (Sanjuán-Sánchez D, 2025)
- “If I did not feel lousy after walking, it would be great for me…” (Sanz‑Baños Y, 2016)
- “For me, the disadvantage is the great effort you make, because, really, it is always an effort…” (Sanz‑Baños Y, 2016)
- “And when I arrive at home at 15:00, I arrive broken, shattered, exhausted...Physically, I am not a person; and mentally, not much...Whereupon, who is able to walk?” (Sanz‑Baños Y, 2016)
- “...because if you are in good mood, you cheer yourself but if you are not, even if you say ‘this is holy glory’...” (Sanz‑Baños Y, 2016)
- “For me, organizing the day is stressful... I am already stressed in the morning.” (Sanz‑Baños Y, 2016)
- “And the fact of having the obligation to go out and walk also causes me some anxiety and distress and makes me feel bad” (Sanz‑Baños Y, 2016)
- “It is more difficult to start over again” (Sanz‑Baños Y, 2016)
- “Things that can beat me down, of course, pain can beat me down, of course. That is undeniable” (Sanz‑Baños Y, 2016)
- “Well I think mood is very important for this, because if you don’t feel in the mood at that time…” (Sanz‑Baños Y, 2016)
- “I can not stand slopes” (Sanz‑Baños Y, 2016)
- “I must not indulge myself” (Takai N, 2022)
- “I stick to swimming because it is the only exercise I can do that doesn’t hurt.” (VanRavenstein K, 2014)

Category 3B: Environmental barriers, costs, time

- “It’s really difficult...when you work and have a family. If you hadn’t had a family, you could have added another 25% to exercising, but if you have a family, they want at least something of you (laughs)” (Larsson A, 2020)
- “I’d ike it to be nearby... Then you know you’ll get it done... It’s much easier when it’s closer...” (Larsson A, 2020)
- “..Um, it gets a lot of worst in extreme temperatures. So-over the winter, there's been times when I've been in education where I haven't turned up, attended once a week or not even-- not at all for several weeks because I can't, um. I can get out of bed -or like walk about the house. But, uh, going out in the cold and walking to the bus stop--just leaves me in agony” (Mayana K.I, 2021)
- "Oh, yeah. oh, yea well, damp weather, damp wet weather when it's cold, bitterly cold. I tend to wear a scarf around my neck “ (Mayana K.I, 2021)
- "And I think it's the weather as well. I know some stupid weather, but I. Yes, I really think it's the weather" (Mayana K.I, 2021)
- "It feels like my body's is bruised, badly bruised, that's probably hard to describe it. And, you know, I think my other half quite
  often, sometimes morning I feel like I've been hit by a bus and that's how I feel. I mean, I mark my pain a lot and sort of I mean, around sort of my shoulders and back, but hands and feet really, particularly when you know the worst areas. They're just feel like say obviously, the weather has made matters even worse" (Mayana K.I, 2021)
- “But because I no longer have a job, I can no longer afford to do that. So, it's been a bit hit and miss the last and then of course we have the lockdown, didn't we? So- Um, obviously if I do get reinstated and-and get some money coming in then I will- I will recommence the classes” (Mayana K.I, 2021)
- “I want to comply, but I see that I can't make it in the morning, that I'm late” (Sanjuán-Sánchez D, 2025)
- "The troubles, the economy, you see that you can't make ends meet. You only get bills" "At the same time you need money” (Sanjuán-Sánchez D, 2025)
- “I have my parents, who are older, who are sick, I always have to go see them. You have to go home and you have your housework to do” (Sanz‑Baños Y, 2016)
- “With a disease like this and to have to work or even take care of family... it’s impossible! Sure walking is great, of course, it is great doing some therapy... Extraordinary! But can we?” (Sanz‑Baños Y, 2016)
- “Being retired. That would give you more time and you can already say: “I will now dedicate myself to me and do these things that this lady is doing” (Sanz‑Baños Y, 2016)
- “That the weather is good…” (Sanz‑Baños Y, 2016)
- “I routinely want to exercise every day. I want to be able to keep up with everything, but I can’t do it either. When I can’t, I quit.” (Sermenli N, 2025)
- “The streets around my home are very crowded during the day, and it is not possible to walk briskly.” (Sermenli N, 2025)
- “...It scares me when it’s dark and there aren’t many people on the street.” (Sermenli N, 2025)
- “For example, when I walk around the block in the evening, my husband says, ‘It’s dark, what are you doing out there? There must be someone (who is dangerous) there,’ or ‘It’s late, don’t go.’ When he says this, my motivation disappears.” (Sermenli N, 2025)
- “I want to go to Pilates (hall) but I can’t. Pilates relaxes me a lot ... the wages are very high.”  (Sermenli N, 2025)
- “I always want exercise to be part of my life, but I can’t fit it into my routine because of other responsibilities, like my husband, my family, and all the other things I need to take care of...” (Sermenli N, 2025)
- “My mind is always on my kids. I take my grandchild to school, chase after him, run here and there...I tell myself, ‘You could find half an hour for yourself if you tried,’ but by then, I’m just too tired.” (Sermenli N, 2025)

Category 3C: Misunderstood by family, employers and clinicians

- "When I went to like, pain therapy and stuff like that it just all involved a lot of stretching and stuff like that. And then I've been recovering for three or four days when I was in so much pain, but no one would listen. They just keep saying do the exercises over and over and no matter how much I did them it was just hurting more and more” (Mayana K.I, 2021)
- “It's like he doesn't even assist like say like coming to do a little a bit of clean. You'll get up to just leave a complete mess that it's silly to have to do. It's just creating more housework than necessary. and You know, I've come I don't have to come back from work and do a massive load of cleaning” (Mayana K.I, 2021)
- “But I don't want to increase the levels because I spent so many years now taking such high doses of tablets because. I. I had blind faith in doctors and GPs. Just take this. This will help keep taking, keep taking it up the dose and if not, it's not a good way to live” (Mayana K.I, 2021)
- “Uh, at the moment I'm unemployed. Um, I was-- Well, I was-- They-they were trying to-- they were trying to medically retire me on the grounds of ill health but and-- they-they actually dismissed me before that process was complete. So, I'm still waiting to see if that will go through. So, at the moment, we're sort of challenging that on the grounds that, um, firstly because I've-I've got a disability and secondly because they just didn't follow the proper processes. But, you know, we just have to wait and see what happens there. So, I kind of seem a bit like stuck between a rock and a hard place, because I can't look for another job because if they reinstate me” (Mayana K.I, 2021)
- “Um, when it happened, it was the first-ever job I'd actually landed. I -got all the way through to the final process, and I had a start date, they were going to send me my uniform. And then I got a letter in the post after the health screening that basically said, um,

"You're not, well because you have chronic pain, we-we're not gonna hire you. And I just-- Uh, I was, um, si-- No, I fibromyalgia- I was-- How old was I? About 17 and that devastated me. It made me feel- like I was-- I couldn't do anything. Um, it's because employers didn't take me on. I had to-- I have had to take legal action in the past because employers -would get to the health screening—and then I declare chronic pain, and they would, um, they wouldn't hire me.” (Mayana K.I, 2021)

- “If you've got a broken leg or a broken arm, that the plaster cast. And you can instantly say something is wrong. But because you're there standing in front of them or going to work or doing whatever you are doing, You're fine. You must be fine. you’re standing there” (Mayana K.I, 2021)
- “I think because it's such a complex and because I had finished work at the time. I think everyone think I was just being lazy or just being difficult. I think that's what they feel. But in the sense of them and in the sense of me, in all honesty, I think I was very paranoid and very insecure. So even if they gave me a normal look at the cutting, though, and I took it as they hated me, and I was vert emotional” (Mayana K.I, 2021)
- “I mean, some people or some friends are basically taking a step away from you because they just did not understand it. And you know what? That's fair enough” (Mayana K.I, 2021)
- “My in-laws even down my brothers, I think because it’s such a complex and because I had finished work at the tie. I think everyone thinks I was just being lazy or just being difficult. Really? I think that’s what they feel” (Mayana K.I, 2021)
- “And a lot of, um, people in my family that don't necessarily understand that can't-can't comprehend that I get so tired after doing so little” (Mayana K.I, 2021)
- “Um, yeah, they try to be. But it's just hard for people to understand because they can't actually see what's wrong with me They know that I'm in pain--but it's like- well, they can't see it so they can't relate to it.. And I- a lot of the time I- they- I get upset because I can't do stuff and I feel like it's putting stuff on the kids that they've got to do what I would normally” (Mayana K.I, 2021)
- “What they were suggesting was that maybe you should try to walk for 10 min” (Montesó-Curto P, 2023)
- “You should try and do this, you should try and do that” (Russel D, 2018)
- "They kicked me out. They threw me out on the street. Nothing... I worked for 4 years without missing a day... and... I never took sick leave and... a month after being on sick leave, they sent me a letter telling me that... if I didn't go back to work, they would fire me. And I, as I was feeling unwell, I didn't go back. Of course, I didn't go back. And they threw me out on the street” (Sanjuán-Sánchez D, 2025)
- "You are there to work. Without even knowing what I had. And I still didn't know what I had. And they don't believe you. They don't believe you. Not even the doctors [Perceives lack of interest on the part of health personnel] Not even the doctors believe you” (Sanjuán-Sánchez D, 2025)
- "But come on, you usually look good. That's why they want us to get to work” (Sanjuán-Sánchez D, 2025)
- “And it was a mistake I made there. Because it turns out that when I told my manager that it was because I hurt my shoulder taking out the box... he said to me: use a spray and it will be like nothing. No...that's nothing. Well, at that moment I should have said to that man: can you take me to the mutual insurance company? Please, I've hurt my shoulder, but I didn't fall.../.../So I went to the Social Security. But if I go to the mutual insurance, then today I would have a salary for the job I was doing. I can't do it anymore. I couldn't even do it now. But as I didn't do it like that... I didn't do it right, so I don't have anything” (Sanjuán-Sánchez D, 2025)
- "It so happens that when employers hear fibromyalgia, they get scared/.../And they reject you a lot. You can never, ever, ever say that you have fibromyalgia. You will never find a job in your life” (Sanjuán-Sánchez D, 2025)
- "You can't go to work they said, and I told them that I wanted to work, and I said: Don't give me sick leave. And they told me: you can't work, with these reports that I receive” (Sanjuán-Sánchez D, 2025)
- “Only I approve of it” (Sanz‑Baños Y, 2016)
- “I’d like to be understood by society, not to be seen like a strange person, that your children and your relatives be well-informed” (Sanz‑Baños Y, 2016)
- “Thus, this entails that you are shutting yourself in your own world... you are giving up meeting people because, the truth is that not everyone understands the situation we are in.” (Sanz‑Baños Y, 2016)
- “No one believes my pain, they think I’m being coy, they call me a hypochondriac... my husband even asks (with a condescending expression) ‘Is it good for you to exercise?’” (Sermenli N, 2025)

Category 3D: Standardized, not personalized plan

- “Exercise classes are really odd because they don't tend to specifically ca-- Like, a lot of the instructors ar-aren't necessarily knowledgeable about--health conditions and they might, yeah. And they don't-- I've had like gym, um, routines where you get set by one of the trainers -and there was one they gave me, and they wanted me to do 50 squats every time -I went to the gym. And my knees are my worst joints. And so, I looked at the guy and was like, "I've-just explained to you I've got chronic pain." (Mayana K.I, 2021)
- “They (providers) have all done it or recommended it because of my weight” (Montesó-Curto P, 2023)
- “The Dr. . . . did. . . the rheumatologist up there. I think my rheumatologist did too and my family internist back home. . . [they] recommended it and there was no implementation on what to do” (Montesó-Curto P, 2023)
- “I have been recommended the exercise bike, and elliptical trainer by professionals of Internal Medicine and rheumatology. I have been visiting a chiropractor for many years” (Montesó-Curto P, 2023)
- “So, there was a fear of that at the time and the anxiety that I am stuck with this pain for the rest of my life. You know, what am I going to do? How am I going to deal with this stuff? . . .they give you exercise programs to relax your muscles, but they don’t really target fibromyalgia. . .If you teach them how to deal with the pain and you have some medication that can work, and you combine the two together then you can really help someone deal with it and to live a very positive lifestyle” (Montesó-Curto P, 2023)
- “I asked the doctor if is there anything at all [I could do] and he got me the 12-week course in the leisure centre, and ... every time I went, I came home and I was put to bed. 3 or 4 days later I dragged myself out of bed to go back to the leisure centre, to try again. I was coming out of the leisure centre every day crying, because the pain was just horrendous. That is the only way I can describe it, absolutely horrendous.” (Russel D, 2018)
- “People with FM can’t follow normal exercise guidelines. They need to start low and go slow.” (VanRavenstein K, 2022)

Theme 4: Facilitators to movement: peer support, empathic relationship and personalize guide

Category 4A: Peer support and benefit of group exercise

- “... I take a crowded train, then I get here; the gym makes me feel better. It’s the contact with my colleagues; it’s very good. I wouldn’t say I feel so good physically, but mentally I feel great.” (Cavaliere A, 2010)
- “Whenever I’m here [in the exercise program], I don’t think about the pain ... you are with your mind on something else ... and you feel better. If you are alone at home ... you are ... it hurts, it hurts” (Beltrán-Carrillo V.J, 2013)
- “I had depression, because my husband died three years ago and this activity ... being with other people helped me to overcome it. I’m always looking forward to the star of the program, so I can have a relationship with the others. Otherwise I don’t have anything else to do… I’ve been left behind from my friends, who have their partners. My children have grown up; they have their lives and have left home… I have more time for myself, what do I want it for?” (Beltrán-Carrillo V.J, 2013)
- “This way I spend some time with the people who come here [exercise program]. Because, I’m very lonely. I live by myself ... there are days I don’t even open the door ... I don’t even open my mouth” (Beltrán-Carrillo V.J, 2013)
- “This is more like a group therapy, here is where they understand you and give you advice.” (Beltrán-Carrillo V.J, 2013)
- “This woman [talking about another fellow] came and ... I thought, “She has the FM face,” what a haggard face. One day we told her, “Are you widowed or something?” and she says, “No, I’m not,” and then I say “Why do you come dressed in black?” She says, “I don’t know” I say, “Then don’t come here dressed in black, ok? It scares me [laughs].” The lady ended up with really cute clothes! That’s why I’m telling you the group in great for us as a therapy.” (Beltrán-Carrillo V.J, 2013)
- “Being with SA brings me to life. One day in the cafeteria she read a manuscript about FM, a story about a sick woman, and I felt identified with it and started crying. She hugged me, she asked me if I had any other problems, she told me to count on her. That same afternoon she called me to see how I was doing.” (Beltrán-Carrillo V.J, 2013)
- “When I was down they got me off the hook. The person who helped me the most told us one day: “This can’t be paid, the only thing we have to do is to do with others what they’ve done for us” ... helping another person doesn’t take anything from you, just the opposite, you gain from it ... and when I do it I feel good about myself ... that’s the main thing.” (Beltrán-Carrillo V.J, 2013)
- “I needed to get out of a group of sick people. Here [in gym] all people have FM like me, but this is a group that tries to get out of it. There are persons in the group that have a very good mood and they spread this joy. Before, in the groups with psychologists, I was fed up of being with people completely down, who only went to complain ... then I went down more and more.” (Beltrán-Carrillo V.J, 2013)
- “..they’re always positive when you arrive...its nice then, you feel that you’re noticed...I have a great need to be noticed...” (Larsson A, 2020)
- “It was nice to know that there are others in this with me, and that they also had a hard time with some poses.” (Lazaridou A, 2019)
- “It is better to do it with others, it makes you do it, you cannot stop it, it is like peer pressure in a good way.” (Lazaridou A, 2019)
- “I was always curious about others, but I didn’t ask.” (Lazaridou A, 2019)
- “It was pretty inspiring. It made me feel normal.” (Lazaridou A, 2019)
- “I did it at home because I had to... I think it is nice to have others around if possible.” (Laàridou A, 2019)
- “I was doing quite badly at that time, so I did not want to make that much contact with anybody, but I think I was mostly in my own world. They were nice, the girls, but we never really talked, no, not like that” (Mannerkorpi K, 2003)
- “The discussion we had when we met have given me so much as I got to know a great deal about my illness that I had not known before. I heard that the others had the same symptoms and I was not alone. That was a positive experience, it was marvellous. I had been thinking that there is something wrong somewhere, I had been thinking I was seriously ill. That it was something…, not that i would die, but I had been wondering what it was, whether I would end up in a wheelchair, or what it all was about” (Mannerkorpi K, 2003)
- “Yes and you could joke a little if somebody limped a bit, or something like that” (Mannerkorpi K, 2003)
- “I think you've had to set up yourself gently. And to be honest, I said on Wednesday that class was such a very gentle class group.” (Mayana K.I, 2021)
- “Um, but I’ve got a group of very close friends who-who are very supportive. And my daughter is actually very supportive because she has clients that she has a long-term chronic condition as well” (MayanaK.I, 2021)
- “Well, I think both that husband spent myself struggle with our own conditions at the moment, which we support each other. You know, we're sympathetic with each other and trying to support each other” (Mayana K.I, 2021)
- “You’re distracted, you’re talking, besides I have to go with someone else, but if I have to go alone, I really do not want to go out. But then, you are talking, you are distracted and I feel like I forget about the pain, the fatigue and all” (Sanz‑Baños Y, 2016)
- “A friend of mine, yes she would do that... if she were in the same situation as me, yes ...a neighbor who is very sporty” (Sanz‑Baños Y, 2016)
- “My husband is always encouraging me to walk ...on vacation, a sister and a brother. And they support me, they cheer me up to come out to walk” (Sanz‑Baños Y, 2016)
- “People like us who have the same disease” (Sanz‑Baños Y, 2016)
- “My friend, who also has fibromyalgia” (Sanz‑Baños Y, 2016)
- “I find more motivation in that moment of solitude...” (Sanz‑Baños Y, 2016)
- “In general, I think that the group doesnot feel like that, like we can not walk…” (Sanz‑Baños Y, 2016)
- “I prefer to go alone, because sometimes I do not feel like talking” (Sanz‑Baños Y, 2016)
- “When a new member arrives, I say to her: ‘To me, the best medicine given to me was to come to the association’. When I came, I first said ‘I will not come anymore’. It gives you a shock. But when we are all equal, we understand each other... A new member comes and you throw yourself into her because she suffers what you have suffered” (Sanz‑Baños Y, 2016)
- “We support each other a lot. Until I met the association, I was isolated, alone and very depressed and as a result of
  knowing the association, seeing young people, people who have been there, experiencing people who have children, who have the same problems as me, I felt supported and this is the people who can encourage and help us, not only to walk, but in general...” (Sanz‑Baños Y, 2016)
- “It’s good to motivate each other. After a while, you might experience pain that others can’t understand, but when the person you’re talking to has gone through it, they can empathize.” (Sermenli N, 2025)
- “I want to be around people who have fibromyalgia and are open to communication and healing. But I don’t want to be around those who have fibromyalgia and are very pessimistic, unhappy, and not solution-oriented in any way because I think they bring people down too much.” (Sermenli N, 2025)

Category 4B: Empathic relationship with healthcare professionals

- “It (the exercise) was hard but still I thought the physiotherapist made sure that we reduced the load if we came into a period where... and that we would do... you didn’t feel forced...to do something you couldn’t do...it was so nice to feel that they were so understanding... I appreciated that...It can be quite difficult if you have someone who...yes, you can do this... because you can’t and then you just go home and feel sad.” (Larsson A, 2020)
- “I loved the instructor... It made a big difference, and I did not want to disappoint the instructor by missing any of the classes.” (Lazaridou A, 2019)
- "The doctor would always tell me: don't suffer, I can't give you sick leave because this illness is not recognised - but as I have
  osteoarthritis - I can give you sick leave and when you are like this, come and I will give you sick leave” (Sanjuán-Sánchez D, 2025)
- “My family doctor is... above all... all I could say is nothing compared to how important he is to me.” (Sanz‑Baños Y, 2016)
- “My doctor supports me and tells me: “You have to go out, also for your Chondromalacia patella, even 10 min but you have to walk”” (Sanz‑Baños Y, 2016)
- “My psychologist supports me in that, too…” (Sanz‑Baños Y, 2016)
- “At first I felt pain when exercising, but I was encouraged by PT and realized I could move more than I thought.” (Takai N, 2022)
- “The PT always encouraged me, saying, ‘You can do it, try one more time.’ That gave me strength.” (Takaii N, 2022)
- “I thought they were very considerate of my pain.” (Takai N, 2022)
- “The PT advised me not to overdo it. That made me feel it was okay to live at my own pace.” (Takai N, 2022)
- “The PT gave me a massage when I was admitted. It was very pleasant and helped me feel at ease.” (Takai N, 2022)
- “Even when the PT was off, another therapist gave me the same care, so I felt reassured.” (Takai N, 2022)
- “The PT talked to me gently, and I felt less anxious.” (Takai N, 2022)
- “They noticed my worries and reassured me often.” (Takai N, 2022)

Category 4C: Need for a personalized guide

- “...I might be able to be there if I got help from someone when I need it, to support me and help me progress slowly and somehow change the exercises a bit, but not too much, and someone to consult with. Some kind of physiotherapist or personal trainer who’s available to consult and who has knowledge about the disease...” (Larsson A, 2020)
- “The yoga videos were huge, they were very helpful... I could see the exercise demonstrated rather than read the instructions on paper.” (Lazaridou A, 2019)
- “IP: I had not realized how effective physical exercise was. Previously I thought that it was more effective to lie down and practise relaxation exercises. But now I have learnt during these meetings that it may in fact be more effective to do something physical. You need both parts, you see. You get rid of that stress (while exercising), and maybe we do exercise far too little if we don’t practise it.” (Mannerkorpi K, 2003)
- “IP: Then (when she was not able to follow the instructions) I only did some movements more slowly or I didn’t do them at all, but I did some other movements, as I tested what I could do and what I could not do. It was the leader who told me that I
  should do this, so if they (the group) were doing movements that I felt were not good for my neck, I did relaxation exercises instead.” (Mannerkorpi K, 2003)
- “There is CBT, and you know, given knowledge of what fibro is and things like that. That was really helpful. And that was probably my turning point, to be honest. it's more acceptance of, you know, I can't do the things that I used to do like going out every weekend dancing. Getting going. Yeah. going to weekends as they dance all day and dance all night. umm going out for a long bike ride. It's known that I can't do those things now, but you know, trying to do what I can.” (Mayana K.I, 2021)
- “It definitely helped because it gave you know, it's so easy to get bogged down in the relentlessness of the cycle of you know pain,work, sleep, no sleep, sleep, pain, work. You know, a few days of. A few days off. And so, you have to have some sort of coping strategy. I Listen to mindfulness. I listen to sort of sleep hypnosis,you know, sometimes you can leave it on all night if necessary. Just try and keep the headphone in just up or distract my mind, my brain. Sometimes it works. Sometimes it doesn't work… You know,it's mindless. There's no clock. You haven't got a, you know, remember anything. And you just dose to sleep, it's a bonus” (Mayana K.I, 2021)
- “I do stretches. . . my own stretches. I learned it here (Mayo) in the program which I am going to implement” (Montesó-Curto P, 2023)
- “I don’t practice exercise. I have tried to do it, but after 10 min of walking, I can’t do it anymore. But even if I do, of course, I live
  in the suburbs, I have 2 girls, I take them up and down, I take them up and down, I take care of them all day long. During the day I do not stop, this says (clock) that today I did 8 km more or less.” (Montesó-Curto P, 2023)
- “Doctors from Pain Relief Unit where I have been going for many years, advise me to walk” (Sanz‑Baños Y, 2016)
- “For me it is better to rest because at some point my feet do not respond” (Sanz‑Baños Y, 2016)
- “If I have someone to push me, it is easier for me” (Sanz‑Baños Y, 2016)
- “I both raise my children and work; I handle everything on my own... I never stop anyway, I am very active (physically)” (Sermenli N, 2025)
- “I would like to know how much exercise I should do, how I should do it, what I should not do” (Sermenli N, 2025)
- “I need a driving force. For example, you... You will say, walk for half an hour or jog for ten minutes today. If you give homework, it can be helpful for me.” (Sermenli N, 2025)
- “At that time, the doctor was saying ‘show your walks (numbers of steps)’, was asking, ’how far did you walk?’. Being under control made me walk, albeit forcibly.” (Sermenli N, 2025)
- “I realized that with proper shoes and PT guidance, I can actually move more than I expected.” (Takai N, 2022)
- “Structured hospital life and regular PT support gave me reassurance” (Takai N, 2022)
- “The PT pointed out the parts of my body where I was tense and taught me how to loosen them.” (Takai N, 2022)
- “They also pointed out my ways of thinking, like ‘I must not indulge myself.’ That was eye-opening.” (Takai N, 2022)
- “They told me, ‘It’s important to listen to your condition and adjust,’ which changed my way of acting.” (Takai N, 2022)
- “They helped me realize the importance of balancing effort and rest.” (Takai N, 2022)
- “At first, I couldn’t move at all, but the PT stayed by me and helped me until I was able to walk.” (Takai N, 2022)
- “I felt secure because they watched over me carefully.” (Takai N, 2022)
- “When I felt resistance at the start of exercise, they accompanied me, and I managed to continue.” (Takai N, 2022)
- “The PT explained clearly, so I could understand what to do.” (Takai N, 2022)
- “They encouraged me at just the right times, which kept me going.” (Takai N, 2022)

*References:*
Beltrán-Carrillo VJ, Tortosa-Martínez J, Jennings G, Sánchez ES. Contributions of a group-based exercise program for coping with fibromyalgia: a qualitative study giving voice to female patients. Women Health. 2013;53(6):612-29. doi: 10.1080/03630242.2013.819399.

Cavaliere, M. L. A., de Abreu Souza, J. M., & de Oliveira Barbosa, J. S. (2010). Representations of the relationship between physical exercise and health for patients with fibromyalgia. Physis, 20(4), 1325.

Larsson A, Feldthusen C, Mannerkorpi K. Factors promoting physical activity in women with fibromyalgia: a qualitative interview study. BMJ Open. 2020;10:e031693.

Lazaridou A, Koulouris A, Dorado K, et al. The impact of a daily yoga programme for women with fibromyalgia. Int J Yoga. 2019;12(3):206-217.

Mannerkorpi K, Gard G. Physiotherapy group treatment for patients with fibromyalgia--an embodied learning process. Disabil Rehabil. 2003 Dec 16;25(24):1372-80. doi: 10.1080/09638280310001616367.

Mayana, KI Association between chronic widespread pain and physical activity behaviour in people with fibromyalgia Degree of Doctor of Philosophy, School of Health and Society, University of Salford, 2021

Montesó-Curto P, Toussaint L, Kueny A, et al. Physical activity and exercise experience in Spanish and US men with fibromyalgia: a qualitative cross-cultural study. Int J Environ Res Public Health. 2023;20(18):6731.

Russell D, Álvarez Gallardo IC, Wilson I, Hughes CM, Davison GW, Sañudo B, McVeigh JG. 'Exercise to me is a scary word': perceptions of fatigue, sleep dysfunction, and exercise in people with fibromyalgia syndrome-a focus group study. Rheumatol Int. 2018 Mar;38(3):507-515. doi: 10.1007/s00296-018-3932-5.

Sanjuan-Sánchez D, Climent-Sanz C, Patiño-Vera M del M, Gea-Sánchez M, Rubí-Carnacea F, Briones Vozmediano E. "Quiero seguir en activo": Estrategias de mujeres que padecen fibromialgia para realizar actividades cotidianas básicas, instrumentales y avanzadas. Arch Prev Riesgos Labor [Internet]. 15 de enero de 2025 [citado 2 de octubre de 2025];28(1):31-53. Disponible en: <https://archivosdeprevencion.eu/index.php/aprl/article/view/405>

Sanz-Baños Y, Pastor MA, Velasco L, et al. To walk or not to walk: insights from a qualitative description study with women suffering from fibromyalgia. Rheumatol Int. 2016;36(8):1135-1143.

Sermenli, N., Sarıtaş, F. & Tonga, E. Perspectives on physical activity among women with fibromyalgia: a qualitative study. J Public Health (Berl.) (2025). <https://doi.org/10.1007/s10389-025-02450-z>

Takai, Noriko ; MARUYAMA, Nobuhiro ; NAKAHARA, Osamu ; SENBA, Emiko ; MIKI, Kenji ; KANEKO, Motoshi ; TAKAHASHI, Noriyo ; FUJITA, Nobuko ; IKEDA, Koji Physical and Mental Effectiveness of a New 3-week Exercise-based Intervention Program for Fibromyalgia Inpatients: Interview Revealed the Importance of Therapists’ Stance, Rigakuryoho Kagaku, 2022, Vol.37(1), pp.45-58

VanRavenstein, Kathryn A. Physical Activity in Women with Fibromyalgia. Diss. Medical University of South Carolina, 2014.

Inizio modulo

Fine modulo
