# Supplementary material for: Perception and lived experience of movement in patients with fibromyalgia: a qualitative systematic review with meta-synthesis and meta-summary
Source: Clin Rheumatol. 2026 Feb 25;45(5):2437–62. doi: 10.1007/s10067-026-08005-1 (PMC13068694; doi:10.1007/s10067-026-08005-1)
Supplement: Supplementary file 6 — Supplementary Material 6 (DOCX 21.9 KB) [file 10067_2026_8005_MOESM6_ESM.docx]

**Supplementary File 6.** Credibility strategies to enhance the trustworthiness and the rigor of the meta-synthesis and meta-summary.

| **IMPLEMENTED STRATEGIES**   - **Multidisciplinary Team of Experts:** A team comprising physiotherapists, nurses, researchers, physicians, and methodologists with expertise in the study’s focus (Fibromyalgia patients’ experience of movement and qualitative research synthesis) collaborated throughout the process. - **Comprehensive Search Strategy:** A systematic search was conducted across seven databases (CINAHL, EMBASE, PsycINFO, MEDLINE, Scopus, SportDiscus, and Web of Science). Additionally, a "berry-picking" method was applied, incorporating techniques such as footnote chasing, citation searching, hand searching, journal runs, author searching, and exploring grey literature. The SPIDER framework (Sample, Phenomenon of Interest, Design, Evaluation, Research type) guided the search for qualitative studies. - **Collaborative Review Process:** Multiple members of the research team participated in all stages of the review, including searching, evaluating, analyzing, and synthesizing data, to ensure rigor and accuracy. - **Involvement of a FM-Experienced Patient:** A patient with lived experience of FM contributed to the project by reviewing the protocol, validating its significance, and emphasizing the review’s importance. The patient also helped interpret qualitative findings, assisted with the metasummary and metasynthesis, and validated the findings to ensure alignment with patient perspectives. - **Regular Team Meetings:** Frequent meetings were held to discuss methodology, data analysis, procedures, and interpretations. A "think aloud" approach facilitated open deliberation, and disagreements were resolved by reaching a consensus. - **Audit Trail:** The entire process of data collection and analysis was thoroughly documented. This included detailing both the planning stages and the specific decisions made during data collection and analysis. Additionally, each phase of the project was recorded, explaining the reasoning behind decisions and the adoption, modification, or abandonment of particular strategies. | | | |
| --- | --- | --- | --- |
| **AUDIT TRAIL** | | | |
| **Meeting** | **Aim** | **Procedure** | **Output** |
| N° 1 | Definition of Research question | - Research problem identification; - Identification of the rational of the study - Identification of the Purpose of the study; - Organization of the time and labour; - Thinking about the possible clinical impact of the study. | - Determination of a research question about perception and experience of movement in patients with Fibromyalgia. |
| N° 2 | Definition of eligibility criteria | - Identification of research parameters; - Identification of topical parameters; - Identification of population parameters; - Identification of temporal parameters; - Identification of methodological parameters. | - Determinations of Inclusion and exclusion criteria; - Identification of the independent reviewers (MC and GG). |
| N° 3 | Definition of search strategy | - Identification of keywords and free terms; - Definition of search strings; - Identification of database; - Identification of berry-picking strategies; - Identification of research limits. | - Determination of keywords and search strings; - Determination of electronic database and berry-picking strategies; - Determination of final research limits; - Identification of independent reviewers (MC and GeG). |
| N° 4 | Quality appraisal assessment | - Need for quality appraisal reflection; - Evaluation and selection of quality appraisal tools; - Research and formulation of the quality appraisal score for the studies. | - Determination of quality appraisal tool; - Determination of quality appraisal score; - Indentification of independent assessors (MC and GiG). |
| N°5 | Certainty assessment | - Need for certainty assessment - Evaluation and selection of certainty assessment tool | - Determination of certainty assessment tool; Indentification of independent assessors (MC and GR). |
| N° 6 | Data extraction and study classification | - Search of the existing extracted data system; - Search of the existing classification system for qualitative studies. | - Determination of final extracted data system; - Determination of final study classification system - Indentification of independent reviewers identification (MM and MS). |
| N° 7 | Data analysis and synthesis | - Reflection about the findings management during the extraction and separation, editing, grouping, abstraction phases; - Reflection about the creation system of codes, categories and themes; - Reflection about the system useful to analyse the findings; - Research about the calculation of the intra-study and inter-study effect size; | - Determination of meta-summary and meta-synthesis process; - Involvement of a patient with FM for the interpretation of qualitative findings - Independent reviewers identification (LP and MS). |
| N° 8 | Review of the outcomes of the eligibility process | - Discussion on the inclusion/exclusion of studies emerged from the search strategy. | - Determination of final inclusion/exclusion of the studies. |
| N° 9 | Review of the outcomes of quality appraisal process | - Discussion regarding the individual item scores of the included studies; - Discussion concerning the overall scores of the included studies. | - Determination of the final quality appraisal scores of the included studies. |
| N° 10 | Review of the outcomes of the data extraction and study classification process. | - Discussion regarding the data extracted from included studies; - Discussion concerning the classification of included studies. | - Determination of the final extracted data; - Classification of the included studies. |
| N° 11 | Review of the outcomes of analysis and synthesis process | - Discussion on managing the findings that arose from the included studies during the subsequent phases of extraction and separation, editing, grouping, and abstraction; - Discussion regarding the developed codes, categories, and themes that emerged from the included studies; - Discussion about the computed intra-study and inter-study effect sizes. | - Determination of the final outcomes of metasummary and meta-synthesis. - Involvement of a patient with FM for validation of patient’s perspective |
| N° 12 | Review of the outcomes of certainty assessment process | - Discussion about confidence in the review findings; - Discussion concerning the overall judgement of the review findings. | - Determination of the final certainty assessment of the review findings. |

**Abbreviations:** CINAHL = Cumulative Index to Nursing and Allied Health Literature; FM=Fibromyalgia
