# Supplementary material for: Perception and lived experience of movement in patients with fibromyalgia: a qualitative systematic review with meta-synthesis and meta-summary
Source: Clin Rheumatol. 2026 Feb 25;45(5):2437–62. doi: 10.1007/s10067-026-08005-1 (PMC13068694; doi:10.1007/s10067-026-08005-1)
Supplement: Supplementary file 7 — Supplementary Material 7 (DOCX 17.1 KB) [file 10067_2026_8005_MOESM7_ESM.docx]

**Supplementary File 7**. Reports excluded and motivations

| **Studies** | **Main reasons for exclusion** |
| --- | --- |
| Antunes MD, Schmitt ACB, Marques AP. Amigos de Fibro (Fibro Friends): development of an educational program for the health promotion of fibromyalgia patients. Prim Health Care Res Dev. 2022 Aug 4;23:e44. doi: 10.1017/S1463423621000773. | Wrong topic |
| Hallberg LR, Bergman S. Minimizing the dysfunctional interplay between activity and recovery: A grounded theory on living with fibromyalgia. Int J Qual Stud Health Well-being. 2011;6(2). doi: 10.3402/qhw.v6i2.7057. | Wrong topic |
| Bourgault P, Lacasse A, Marchand S, Courtemanche-Harel R, Charest J, Gaumond I, Barcellos de Souza J, Choinière M. Multicomponent interdisciplinary group intervention for self-management of fibromyalgia: a mixed-methods randomized controlled trial. PLoS One. 2015 May 15;10(5):e0126324. doi: 10.1371/journal.pone.0126324. | Wrong topic |
| Arfuch VM, Caballol Angelats R, Aguilar Martín C, Gonçalves AQ, Carrasco-Querol N, González Serra G, Sancho Sol MC, Fusté Anguera I, Friberg E, Berenguera A. Patients' Lived Experience in a Multicomponent Intervention for Fibromyalgia Syndrome in Primary Care: A Qualitative Interview Study. Int J Environ Res Public Health. 2022 Oct 15;19(20):13322. doi: 10.3390/ijerph192013322. | Wrong topic |
| Blázquez A, Guillamó E, Javierre C.. Preliminary experience with dance movement therapy in patients with chronic fatigue syndrome. The Arts in Psychotherapy. 2010;37(4), 285–292. https://doi.org/10.1016/j.aip.2010.05.003 | Wrong population |
| Steihaug S. Women's strategies for handling chronic muscle pain: a qualitative study. Scand J Prim Health Care. 2007 Mar;25(1):44-8. doi: 10.1080/02813430601016944*.* | Wrong population |
| Unal E, Karaca NB, Saldirdak GA, Albayrak H, Ozcadirci A, Nacar NE, Tufekci O, Buran S, Kiraz S, Cankurtaran O. Investigation of the Effectiveness of a Biopsychosocial-Based Exercise Approach in Rheumatic Diseases: A Mixed Methods Research With Patients' Perspectives. J Eval Clin Pract. 2025 Feb;31(1):e70033. doi: 10.1111/jep.70033. | Wrong population |
| Karper WB, Hopewell R, Hodge M. Exercise program effects on women with fibromyalgia syndrome. Clin Nurse Spec. 2001 Mar;15(2):67-73; quiz 74-5. doi: 10.1097/00002800-200103000-00009. | Wrong study design |
| Merry G, Cairns M. The AM-FM Study (Aquatic physiotherapy Management in FibroMyalgia): Exploring Patients' Perceptions About Aquatic Exercise in the Treatment and Management of Fibromyalgia. The Journal of Aquatic Physical Therapy (JAPT). 2023; 31(1), 11-19. https://doi.org/10.1097/PXT.0000000000000024 | Wrong study design |
| Pastor M Á, López-Roig S, Sanz Y, Peñacoba C, Cigarán M, Velasco L, Lledò A, Écija C. Walking as physical exercise in Fibromyalgia: An elicitation study from the Theory of Planned Behavior. Anales de Psicología. 2015;31(2), 443-446. |  |
|  |  |
| Martínez-Amat A, Hita-Contreras F, Latorre-Román PA, Gutierrez-López Mde L, García-Pinillos F, Martínez-López EJ. Association of the weekly practice of guided physical activity with the reduction of falls and symptoms of fibromyalgia in adult women. J Strength Cond Res. 2014 Nov;28(11):3146-54. doi: 10.1519/JSC.0000000000000503. | Wrong study design |
